# Evaluation of the Drug–Drug Interaction Potential of Cannabidiol Against UGT2B7-Mediated Morphine Metabolism Using Physiologically Based Pharmacokinetic Modeling

**DOI:** 10.3390/pharmaceutics16121599

**Published:** 2024-12-16

**Authors:** Shelby Coates, Keti Bardhi, Bhagwat Prasad, Philip Lazarus

**Affiliations:** 1 Department of Pharmaceutical Sciences, College of Pharmacy and Pharmaceutical Sciences, Washington State University, 412 E Spokane Falls Blvd., Spokane, WA 99202, USA; 2 Division of Quantitative Molecular Biosciences, Department of Pharmaceutical Sciences, School of Pharmacy and Pharmaceutical Sciences, SUNY University at Buffalo, 160 Hayes Rd., Buffalo, NY 14215, USA

**Keywords:** physiologically based pharmacokinetic modeling (PBPK), UDP-glucuronosyltransferase (UGT), cannabidiol, cannabinoid, morphine, IVIVE, drug–drug interaction (DDI), cannabis, opioid

## Abstract

**Background:** Morphine is a commonly prescribed opioid analgesic used to treat chronic pain. Morphine undergoes glucuronidation by UDP-glucuronosyltransferase (UGT) 2B7 to form morphine-3-glucuronide and morphine-6-glucuronide. Morphine is the gold standard for chronic pain management and has a narrow therapeutic index. Reports have shown that chronic pain patients have increasingly used other supplements to treat their chronic pain, including cannabidiol (CBD). Up to 50% of chronic pain patients report that they co-use cannabis with their prescribed opioid for pain management, including morphine. Previous work has shown that cannabidiol is a potent inhibitor of UGT2B7, including morphine-mediated metabolism. Co-use of morphine and CBD may result in unwanted drug–drug interactions (DDIs). **Methods:** Using available physiochemical and clinical parameters, morphine and CBD physiologically based pharmacokinetic (PBPK) models were developed and validated in both healthy and cirrhotic populations. Models for the two populations were then combined to predict the severity and clinical relevance of the potential DDIs during coadministration of both morphine and CBD in both healthy and hepatic-impaired virtual populations. **Results:** The predictive DDI model suggests that a ~5% increase in morphine exposure is to be expected in healthy populations. A similar increase in exposure of morphine is predicted in severe hepatic-impaired populations with an increase of ~10. **Conclusions:** While these predicted increases in morphine exposure are below the Food and Drug Administration’s cutoff (1.25-fold increase), morphine has a narrow therapeutic index and a 5–10% increase in exposure may be clinically relevant. Future clinical studies are needed to fully characterize the clinical relevance of morphine-related DDIs.

## 1. Introduction

Morphine is an opioid analgesic that is commonly used to treat moderate to severe chronic pain [1,2]. Morphine is still considered to be the “gold standard” in pain management care and in other medical settings [3]. Metabolism of morphine occurs primarily in the liver, where morphine undergoes glucuronidation by UDP-glucuronosyltransferase (UGT) 2B7 to form morphine-3-glucuronide and morphine-6-glucuronide [4,5]. UGT2B7-meditead metabolism is the primary metabolic pathway of morphine [6]. Morphine-3-glucuronide is not pharmacologically active; however, it is associated with negative side effects of morphine [7,8]. In contrast, morphine-6-glucuronide is pharmacologically active and is thought to contribute to morphine’s efficacy [9,10].

Cannabidiol (CBD), a major cannabinoid found in the cannabis plant, is available ‘over-the-counter’, and is legal within the United States. Its synthetic form, Epidiolex^TM^, is used for the treatment of Dravet’s syndrome, Lennox–Gastaut syndrome, and tuberous sclerosis complex [11]. CBD is commonly used to self-treat several ailments including pain, anxiety, and depression [12,13]. The combination of opioids and cannabis is increasingly common [14,15,16], with up to 40% of chronic pain patients supplementing their opioid pain regimen with cannabis, including CBD [17]. Since morphine is a narrow therapeutic index drug, it is important to investigate the potential pharmacokinetic DDIs that may arise from concomitant use of morphine and CBD.

Previous in vitro studies have shown that major cannabinoids and their metabolites inhibit both CYP and UGT enzymes, including morphine UGT2B7-mediated glucuronidation [18,19,20,21,22,23]. In vitro to in vivo extrapolation (IVIVE) using static mechanistic modeling was performed to determine the potential for a drug–drug interaction (DDI) to occur in vivo with coadministration of morphine and individual cannabinoids [21]. While static mechanistic modeling of potential DDIs is useful, it only takes into consideration a single inhibitor concentration (C_max_), and thus, only predicts the potential for a DDI based upon the worst-case scenario. Dynamic modeling, such as physiologically based pharmacokinetic (PBPK) modeling, is increasingly used to predict DDIs and has been utilized in drug approval applications waivers by the FDA [24,25]. Dynamic modeling is the preferred method to investigating potential DDIs as it allows for both the substrate and inhibitor concentration–time profiles to be considered when predicting a DDI. Furthermore, static modeling only allows for a single inhibitor to be modeled, as compared to PBPK where multiple inhibitors can be examined against one substrate simultaneously. This generates a more accurate prediction of an in vivo scenario of the potential DDI, especially for cases where a drug is taken with a drug or natural product that contains multiples of compounds like cannabis.

Previous research has shown that CBD can inhibit numerous drug-metabolizing enzymes in vitro including cytochrome P450 (CYP)s, UGTs, and carboxylesterases (CES), with static and dynamic modeling indicating the potential for a DDI in vivo [18,19,20,21,22,23], and these interactions may be associated with disease state. Taylor et al. [26] found that people with mild, moderate, and severe hepatic impairment had up to 4-fold higher exposure (AUC and C_max_) to CBD compared to healthy individuals. Furthermore, Watkins et al. [27] found that CBD can be hepatoxic as seen by elevated levels of alanine aminotransferase after 750 mg B.I.D. of CBD. After administration of Epidiolex™, this elevation in alanine aminotransferase elevation is dose-dependent from 10 to 20 mg/kg/day [28]. Due to CBD’s safety profile and inherent physiochemical properties (high Log P), it is increasingly important to examine the potential for CBD-mediated DDIs. Based upon previous research, the objective of the current study was to use dynamic physiologically based pharmacokinetic (PBPK) modeling to further evaluate potential DDIs between CBD and morphine by developing and validating both morphine and CBD PBPK models and assess the magnitude of potential DDIs between CBD and morphine in both healthy and cirrhotic populations.

## 2. Materials and Methods

### 2.1. PBPK Model Development and Validation

All PBPK models were developed using Simcyp software version 23.1 (Simcyp, Sheffield, UK). Input parameters including physiochemical properties, blood binding, and pharmacokinetic parameters used to simulate the pharmacokinetics of each drug are listed in Supplemental Table S1 (morphine) and Supplemental Table S2 (CBD). Morphine’s clearance profile was based on the model of Emoto et al. [29] and the advanced dissolution, absorption, and metabolism (ADAM) model for morphine based upon the model from Uchaipichat et al. (2022) [30]. The morphine model was developed and validated using the healthy adult population within Simcyp. A full PBPK model based upon Rodgers and Rowland’s method [31,32] was used in simulations to predict morphine distribution. A permeability-limited liver model was used to describe morphine distribution within the liver [29,33,34]. This model included OCT1 transport in the liver and UGT2B7-mediated metabolism to M3G and M6G using optimized enzyme and transporter kinetics [29,35,36]. To model controlled-release (CR) formulations of morphine, the release profile was taken from previous studies [37] and incorporated into the ADAM model. The model was also validated in a hepatically impaired population for both IV and oral formulations using the existing Simcyp model (Child–Pugh C) for severe hepatic impairment (HI). Brain tissue concentration was predicted after validation using cerebrospinal fluid (CSF) concentrations to model morphine exposure within the brain. P-glycoprotein CL_int_ was taken from Verscheijden et al. [38] and incorporated into a permeability-limited brain model. The passive permeability-surface area product (PSB) on the blood–brain barrier (BBB) and the passive permeability-surface area product on the blood–CSF barrier (PSC) were optimized to recapitulate the observed CSF morphine concentrations, where the PSC is expected to be half of the PSB (half the surface area) [39]. Morphine CSF concentration was used as a surrogate for morphine brain tissue concentration during model development due to lack of available clinical data of morphine concentration in the brain. The PBPK model development for CBD was based upon Bansal et al. [40] and can be found in the Supplementary Methods. Simulations were conducted using the Simcyp healthy volunteers’ population, with a minimum of 400 subjects per trial; number of subjects, age, sex, route of administration, dose, and fed vs. fasted state were identical to those in the corresponding clinical study. Model predictive performance was evaluated by comparing the simulated area under the concentration–time curve (AUC) and maximum plasma concentration (C_max_) to the observed in vivo data that were used as validation sets (not training sets). The model was validated if the predicted AUC and C_max_ values were within 0.5- to 2-fold the range of observed in vivo data. The model was further analyzed visually by comparing if the observed data points fell within the 95% confidence intervals (CIs) of the simulated concentration–time curve (WebPlotDigitizer v4.7; https://automeris.io/WebPlotDigitizer; URL accessed on 1 September 2023). Model validation was also statistically analyzed by determining the mean relative deviation (MRD) and geometric mean fold error (GMFE) of both the AUC and C_max_ predicted-to-observed ratios (Supplementary Methods).

### 2.2. DDI Prediction and Model Validation

Sensitivity analysis was performed to evaluate predictive model performance by decreasing the K_i_ values of CBD against morphine glucuronidation by 1- to 10-fold. It is expected that with decreasing K_i_ values the AUCR would increase.

DDI trials were simulated with 400 subjects (20 subjects × 20 trials) in healthy adults with an equal proportion of males and females, and an age range of 20–40 years. CBD oral doses (1500 mg twice daily) were administered for 7 days, while morphine was set as single IV and oral doses, which were co-administered with CBD on day 6. The IV, oral solution, immediate-release (IR) tablets, and controlled-release (CR) morphine doses were set at 3.76 mg, 11.7 mg, 15.2 mg, and 22.6 mg, respectively. The magnitude of the DDI is presented as the morphine AUC ratio when co-administered with CBD. DDI trials were also simulated with severe hepatic impairment with 400 subjects (20 subjects × 20 trials) with an equal proportion of females and an age range of 20–40 years.

## 3. Results

### 3.1. Morphine PBPK Model Validation After IV and Oral Administration

**Figure 1:**
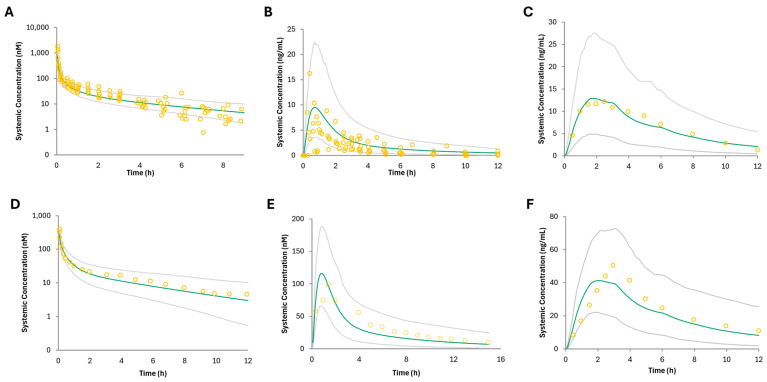
PBPK model of predicted and observed morphine plasma pharmacokinetic profiles after (**A**) IV (Lotsch et al., 2002) [41], (**B**) IR oral (Hoskin et al., 1989) [42], and (**C**) CR (Kotb et al., 2005) [43] oral administration in healthy adults and (**D**) IV (Hasselstrom et al., 1990) [44], (**E**) IR oral (Hasselstrom et al., 1990) [44], and (**F**) CR oral (Kotb et al., 2005) [43] administration in adults with cirrhosis. Data points are the observed values, and the green line represents the model’s predicted morphine plasma concentration–time profile. Gray lines indicate the 5th–95th percentile of the virtual population.

The PBPK model—predicted morphine plasma concentration—time profiles following IV, IR, and CR administration were comparable to the observed in vivo data used as training and validation datasets (Figure 1; Supplemental Figures S1 and S2). The mean GMFE and MRD predicted-to-observed AUC and C_max_ ratios were below the 2-fold cut off for all formulations (Supplemental Table S3). The mean GMFE and MRD predicted-to-observed AUC ratios for IV were 1.71 and 1.65, respectively (Supplemental Table S3). The mean GMFE and MRD predicted-to-observed AUC ratios were 2.00 and 1.96, and 1.96 and 1.95 for predicted-to-observed C_max_ ratios, for IR formulations, and were 2.00 and 1.70, and 1.95 and 1.42, for predicted-to-observed AUC and C_max_ ratios, respectively, for CR formulations (Supplemental Table S3). These results indicate successful development of a PBPK model for morphine after IV and oral administration that can simulate clinically observed pharmacokinetics of morphine. PBPK models accurately predicted morphine CSF concentrations after IV administration (Supplemental Figure S3) and GMFE/C_max_ and MRD/C_max_ ratios were below the 2-fold cut off (1.24 and 1.02, respectively; Supplemental Table S3). The predicted morphine brain concentration–time profile following IV administration is also shown and is slightly higher (21.32 ng/mL) than the predicted and observed morphine CSF concentrations (Supplemental Figure S3), suggesting that the CSF concentration may be a good surrogate for brain tissue concentrations.

### 3.2. CBD PBPK Model Validation After IV and Oral Administration

**Figure 2:**
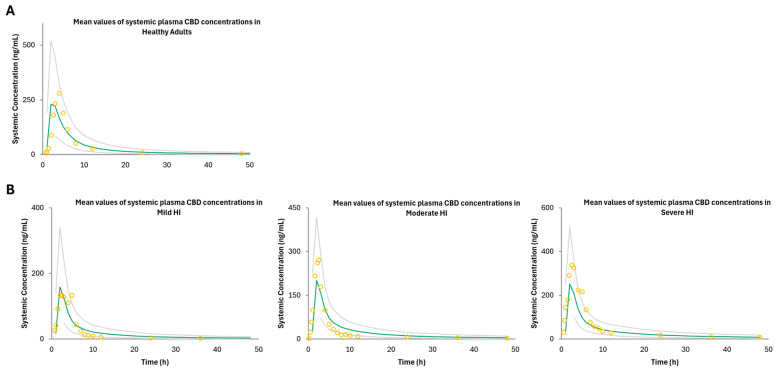
PBPK model of predicted and observed CBD plasma pharmacokinetic profiles after (**A**) oral administration in healthy adults (Taylor et al., 2018) [45] and (**B**) oral administration in adults with cirrhosis (Taylor et al., 2019) [26]. Data points are the observed values, and the green line represents the model-predicted morphine plasma concentration–time profiles. Gray lines indicate the 5th–95th percentile of the virtual population.

**Figure 3:**
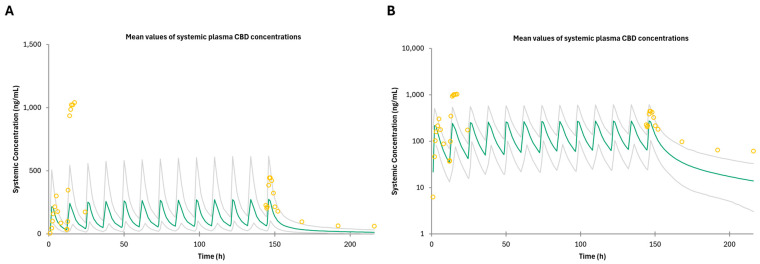
PBPK model of predicted and observed CBD plasma pharmacokinetic profiles after oral administration of 1500 mg twice daily for 7 days in healthy adults plotted on a (**A**) non-log, and (**B**) log scale. Data points are the observed values, and green the line represents the model-predicted CBD plasma concentration–time profile. Gray lines indicate the 5th–95th percentile of the virtual population.

The PBPK model—predicted CBD plasma concentration—time profiles following IV and oral administration in both the fasted and fed states were comparable to the observed in vivo data used as a training dataset (Figure 2, Supplemental Figures S4 and S5). The GMFE and MRD predicted-to-observed AUC and C_max_ ratios were below 2, indicating an acceptable model (see Supplemental Table S4). The mean GMFE and MRD predicted-to-observed AUC ratios were 1.91 and 1.2, respectively, for IV (Supplemental Table S3). The mean GMFE and MRD predicted-to-observed AUC and C_max_ ratios were 1.87 and 1.49, and 1.59 and 1.35, respectively, for the fasted state, and 1.33 and 1.06, and 1.80 and 1.24, respectively, for the fed state, respectively (Supplemental Table S4). These results indicate successful development of a PBPK model for CBD after IV and oral administration that can simulate clinically observed pharmacokinetics of CBD. The CBD predicted plasma concentration–time profile following multiple doses of oral administration in the fasted state was comparable to the observed in vivo data (Figure 3). The GMFE and MRD predicted-to-observed AUC and C_max_ ratios were below the 2-fold cut off (Supplemental Table S4). The GMFE and MRD predicted-to-observed AUC and C_max_ ratios were 1.67 and 1.22, and 2.00 and 1.40, respectively, as compared to the observed AUC and C_max_ measured on days 1 and 7 after multiple dose administration of CBD to healthy subjects (Supplemental Table S4).

### 3.3. PBPK Model Validation in Hepatic-Impaired Adult Populations

The PBPK model—predicted morphine plasma concentration—time profiles following IV and oral administration in cirrhotic populations (Child–Pugh C) were comparable to the observed in vivo data used for this analysis (Figure 1). The CR severe hepatic impairment model GMFE and MRD were greater than 2, indicating underprediction of both the AUC and C_max_; the model is still considered acceptable based upon the other acceptance criteria (Supplemental Table S3). The mean GMFE and MRD predicted-to-observed AUC ratios for IV were 1.68 and 1.12, the mean GMFE and MRD predicted-to-observed AUC and C_max_ ratios for IR formulations were 2.30 and 1.35 (AUC) and 1.60 and 1.1 (C_max_), and the mean GMFE and MRD predicted-to-observed AUC and C_max_ ratios for CR formulations were 3.16 and 1.76 (AUC) and 2.21 and 1.32 (C_max_) (Supplemental Table S3). The PBPK model—predicted CBD plasma concentration—time profiles following oral administration in mild, moderate, and severe hepatic impairment were comparable (> 0.5-fold of the observed AUC and C_max_) to the observed in vivo data used (Figure 3; Supplemental Table S4). The GMFE and MRD predicted-to-observed AUC and C_max_ ratios for mild HI were 1.79 and 1.16 (AUC), 2.17 and 1.30 (C_max_); the GMFE and MRD predicted-to-observed AUC and C_max_ ratios for moderate HI were 1.30 and 1.03 (AUC) and 2.86 and 1.61 (C_max_); and the GMFE and MRD predicted-to-observed AUC and C_max_ ratios for severe HI were 1.93 and 1.20 (AUC) and 2.26 and 1.33 (C_max_), with a combined GMFE and MRD of 1.70 and 1.27 for AUC, and 2.43 and 1.85 for C_max_, respectively (Supplemental Table S4). Although the C_max_ GMFE for mild, moderate, and severe HI is greater than 2, indicating under prediction of the observed C_max_ compared to the simulation, the model is still considered acceptable based upon the other acceptance criteria and similar findings in Bansal et al.’s HI models [40].

### 3.4. PBPK Model for DDI Simulation with Coadministration of Morphine and CBD

The validated PBPK models for morphine and CBD were combined to predict the magnitude of the potential DDI arising from inhibition of morphine glucuronidation by CBD in a virtual healthy population and severe HI population. Due to the lack of clinical data investigating this potential DDI for model validation, sensitivity analysis was employed to evaluate model robustness; decreasing the K_i_ value (1–10-fold) may increase the ratio of simulate morphine AUC. As shown in Supplemental Figure S6, reduced K_i_ values for CBD against morphine glucuronidation in healthy subjects resulted in an expected increase in the simulated morphine AUC in both IV and oral administration models.

Based upon virtual trials with healthy subjects, coadministration of CBD (1500 mg twice daily for 7 days) and 11.7 mg of morphine (oral solution) or 15.2 mg of IR morphine leads to a mild increase in morphine exposure and C_max_ (6%; Table 1; Supplemental Figure S7). Based upon virtual trials with subjects with severe hepatic impairment, coadministration of CBD (1500 mg twice daily for 7 days) and either 11.7 mg of morphine (oral solution) or 15.2 mg of IR morphine also leads to a mild increase in morphine exposure and C_max_ (7% and 6%, respectively; Table 1; Supplemental Figure S7). In virtual trials with healthy subjects, coadministration of CBD (1500 mg twice daily for 7 days) with 22.6 mg of CR oral morphine led to an AUCR and C_max_ ratio of 1.04 and 1.05, respectively, while the trials in an adult population with severe hepatic impairment with coadministration of CBD saw an AUCR and C_max_ ratio of 1.06 (Table S1).

**Figure 4:**
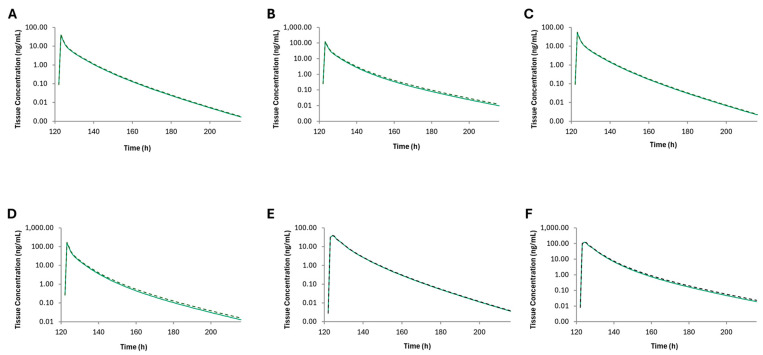
Brain tissue concentrations of morphine after oral administration of (**A**) oral solution (11.7 mg) in healthy adults, (**B**) oral solution (11.7 mg) in adults with severe hepatic impairment, (**C**) immediate-release tablet (15.2 mg) in healthy adults, (**D**) immediate-release tablet (15.2 mg) in adults with severe hepatic impairment, (**E**) controlled-release tablet (22.6 mg) in healthy adults, and (**F**) controlled-release tablet (22.6 mg) in adults with severe hepatic impairment. Shown are the mean brain morphine concentrations with (black dashed line or without (green line) cannabidiol over time.

**Figure 5:**
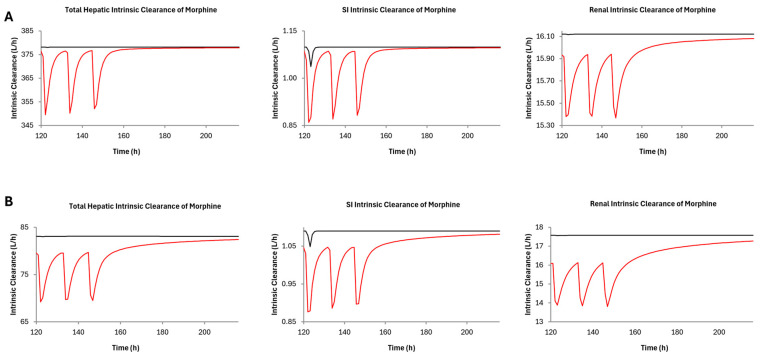
Morphine intrinsic clearance (CL_int_) after CBD administration (1500 mg twice daily for 7 days) with 15.2 mg of immediate-release morphine in (**A**) healthy and (**B**) hepatically impaired adults. The red and black lines represent morphine CL_int_ with and without the presence of CBD, respectively. There was a 7.6, 21.8, and 4.8% decrease in hepatic, small intestine, and renal CL_int_, respectively, in healthy adults, and a 17.2, 19.6, and 21.4% decrease in hepatic, small intestine, and renal CL_int_, respectively, in adults with cirrhosis.

Predicted morphine brain tissue concentrations after concomitant administration of CBD and morphine in healthy adults and adults with cirrhosis increased by 6% and 9% after administration of each formulation of morphine (Figure 4). Furthermore, the active metabolite, morphine-6-glucuronide, concentrations decreased after administration of CBD. Based upon (intrinsic organ clearance) CL_int_ in the liver, kidney, and the gut—the DDI has the largest impact in the small intestine (Figure 5) in healthy populations with a decrease in CL_int_ by 21.8%, whereas the largest decrease in CL_int_ occurred in the kidneys (21.4%) in HI populations.

## 4. Discussion

Our previous in vitro studies have found that major cannabinoids, including CBD and their metabolites, inhibit UGT2B7-mediated metabolism of multiple substrates, including zidovudine, oxazepam, and morphine [19,20,21]. Utilizing static mechanistic modeling as recommended by the FDA [21,46], it was found that major cannabinoids, specifically CBD, could potentially cause DDI when co-administered with morphine (AUCR ≥ 1.25). However, results from the present study using PBPK models indicate less dramatic inhibition of morphine UGT2B7-mediated metabolism by CBD. While only a 5–10% increase in morphine exposure was predicted with PBPK models in both healthy and cirrhotic populations, this increase in morphine exposure may still be clinically relevant. Like all opioids, morphine has a narrow therapeutic index [47], and a major adverse side effect of morphine use is respiratory depression. Thus, while a 5–10% increase may not be classified as a clinically relevant DDI pharmacokinetically as defined by the FDA [46], such an increase could lead to increases in morphine concentrations in the brain that may lead to changes in pharmacodynamic effects. This may be clinically relevant and could lead to increased respiratory depression or overdose, varying between individuals. Therefore, morphine dose adjustments or adjustments in the frequency of morphine administration may still be necessary during coadministration with CBD to ensure patient safety. Furthermore, individuals who are taking morphine to manage their chronic pain are likely part of a patient population that takes multiple medications simultaneously, further increasing the risk for DDI and the need for dose adjustments as the effects of other concomitant drugs on this interaction should be considered. Therefore, it is prudent for clinicians to monitor patients who concomitantly use CBD and morphine to ensure that morphine plasma concentrations are within the therapeutic window.

Results from the present study are similar to Uchaipichat et al., where PBPK modeling was used to determine the potential for a DDI between morphine and diclofenac and S-naproxen [30]. Minimal increases in morphine exposure were observed with coadministration of S-naproxen (1–13%) [30,46]. However, they noted that a mild increase in morphine exposure may still be clinically relevant due to the narrow therapeutic window of morphine [30].

The CBD models used to simulate morphine exposure in mild, moderate, and severe HI varied from the model described in Bansal et al. [40]. The previously reported CBD model incorporated proteomic data changes in the total abundance of both UGT1A9 and UGT2B7 to model CBD exposure in the three HI populations (Child–Pugh A-C) within Simcyp as Simcyp V20 had yet to incorporate UGT total abundance into models [40]. However, the current version of Simcyp V23 does incorporate UGT abundance changes in Child–Pugh A-C models. In the present study, the CBD HI models described previously [40] were confirmed by comparing the default Child–Pugh A and B Simcyp model which incorporates UGT enzyme abundance vs. the values used from El-Khateeb et al., improving recapitulation of observed data (see Figure 3) [48]. However, the abundance changes previously reported were used for the Child–Pugh C model as the predicted exposure of CBD based on those abundance values best fit the observed data (Figure 3). The default Child–Pugh C model incorporating UGT2B7 abundance values best described the morphine cirrhotic population data and was used for DDI modeling.

Hepatic impairment appeared to have little effect on the potential DDI between morphine and CBD. Uchaipichat et al. also found similar results where the increase in morphine exposure after administration with the perpetrator drug was less in cirrhotic populations compared to healthy volunteers [30]. This is contradictory to previous pharmacokinetic studies where morphine exposure increased, and the hepatic extraction ratio decreased in cirrhotic patients [44,49,50,51]. However, one study found no difference in morphine exposure and hepatic extraction in cirrhosis patients compared to healthy controls [52]. The difference between these studies could be due to the severity of liver cirrhosis in the patients in each of these studies.

Hasselström et al. found that dosage reduction is needed in cirrhotic patients who are prescribed morphine to avoid toxicity and adverse side effects [44] and this will be increasingly important if the patient is also taking CBD simultaneously not only due to a potential DDI but also due to the already compromised liver state and that chronic CBD administration leads to hepatoxicity [27]. The DDI simulations in cirrhotic patients in the present study saw a predicted morphine C_max_ of 49 ng/mL after coadministration of CBD. Hasselström et al. saw a morphine C_max_ of 29.4 ng/mL in cirrhotic patients and noted numerous side effects seen in the participants [44]. This suggests that a morphine concentration of 49 ng/mL would be even more likely to cause adverse side effects and necessitate dosage adjustments when taking both morphine and CBD. Furthermore, studies indicate that up to 30% of morphine glucuronidation occurs extrahepatically in cirrhotic patients compared to 10% in healthy volunteers [44,49]. This suggests that liver cirrhosis may not contribute to the potential DDI between morphine and CBD and that compensatory mechanisms are present to accommodate morphine metabolism when liver cirrhosis is present. This was observed in the DDI models in severe hepatic impairment in the present study—the renal contribution increased and subsequent renal CL_int_ decreased by 21.4% in the presence of CBD (Figure 5).

CBD exposure was also found to increase in mild, moderate, and severe HI patients [26]. The lack of increased morphine exposure in the HI virtual population in the presence of CBD is likely due to the CBD K_i_ value against UGT2B7. While the exposure of both morphine and CBD increased in cirrhotic populations, the overall inhibitory effect remained similar to that observed in healthy populations due to the K_i_ value being within physiological levels of CBD (though not for long periods of time based upon simulated CBD plasma concentration–time curves; Figure 3). It is likely that CBD is quickly metabolized by multiple enzymes (Supplemental Table S2), resulting in the concentrations of CBD being high enough to meet the K_i_ to exert an inhibitory effect on UGT2B7 only briefly.

Within the clinical studies used to develop and validate the morphine–CBD DDI models presented in this study, the mean morphine plasma concentration was 8.7 ng/mL and the mean simulated morphine concentration after coadministration with CBD was 14.8 ng/mL. The mean reported therapeutic window for morphine plasma concentrations is 9–80 ng/mL [53]. This suggests that toxicity and adverse side effects are seen when morphine plasma concentrations exceed 80 ng/mL [54]. However, as described above, Hasselström et al. saw adverse side effects in morphine patients with a plasma concentration of 29 ng/mL [44]. This suggests that morphine-associated side effects can occur within the therapeutic window and vary between individuals due to several factors including opioid tolerance and natural steady-state concentrations. Thus, a mild increase in morphine exposure may still lead to adverse side effects due to interindividual variability in response to morphine. Furthermore, these studies do not report the brain tissue concentrations that correspond to the therapeutic window plasma concentrations of morphine.

Morphine has two primary metabolites, morphine-3-glucuronide and morphine-6-glucuronide. The models presented in this study did not predict the exposure of either metabolite in the presence of CBD. However, these models were able to describe a decrease in formation of both morphine-3-glucuronide and morphine-6-glucuronide due to the inhibition of UGT2B7. It has been reported that morphine-6-glucuronide contributes to the analgesic efficacy of morphine [8,9,10], and subsequent inhibition of metabolite formation would decrease its contribution to analgesia. However, the subsequent increase in morphine due to inhibition of UGT2B7-mediated metabolism to morphine-6-glucuronide may ameliorate the loss of contribution of analgesia from morphine-6-glucuronide. This requires future studies incorporating pharmacodynamic effects into the model. Furthermore, the decrease in morphine-3-glucuronide formation would be predicted to be beneficial as it is known to be neuroexcitatory and cause adverse side effects [7,8].

The disconnect between the current results and previous findings using static modeling may be because static modeling only considers a single concentration of the inhibitor (C_max_) and thus is a worst-case scenario, whereas with PBPK modeling, the concentration of the inhibitor over time is considered and gives a more realistic prediction of the DDI potential between two or more compounds. PBPK modeling also allows prediction of tissue concentrations (i.e., liver or intestine) of the inhibitor to determine whether a perpetrator drug reaches high enough concentrations to cause a DDI, whereas static modeling is based on a single systemic concentration of an inhibitor (i.e., worst-case scenario).

The current study has several limitations. It is important to consider morphine and CBD use in a broader context where misuse may occur. For example, the DDI models reported in the present study may not accurately predict and assess DDI risk in individuals who are taking higher CBD doses. The current study performed predictive DDI models with a single week of multiple CBD doses that are higher than current dosing regimen strategies for Epidiolex™ [11], as pharmacokinetic studies for CBD at clinically relevant doses are not available. Therefore, the clinical data used for the present study may not represent the scenario of chronic CBD administration that individuals may use in terms of self-medicating doses of CBD or frequency of doses. Additional DDI simulations utilizing larger doses of CBD are needed to investigate the potential implications of high dose CBD-induced drug interactions with morphine. In addition, the models presented in this study did not incorporate the inhibitory effects of CBD’s metabolites that have much larger exposures as compared to CBD itself. In previous studies, it was found that 7-OH-CBD also inhibited UGT2B7-mediated morphine metabolism [21]. Furthermore, 7-OH-CBD exposure is comparable to CBD exposure in healthy patients [54]. Thus, the potential for a DDI between CBD and morphine may be more severe than predicted by the data from the present study. A model incorporating CBD’s metabolites would better characterize the inhibitory effect of concomitant usage of CBD and morphine. Also, the model did not incorporate the potential pharmacodynamic effects an increase in morphine exposure would have in either patient population. Future models can be developed to investigate the pharmacodynamic effects of inhibition of morphine UGT2B7-mediated metabolism by CBD and its major metabolites. Furthermore, only one study was available that measured CSF concentrations after morphine IV administration; therefore, the models presented in the present study could not be validated with another study for IV administration or in oral administration.

In summary, the present study is the first to investigate the potential DDI between morphine and CBD using PBPK modeling in two populations. CBD was shown to mildly increase morphine exposure (5–10%) in both healthy and cirrhotic populations after multiple doses of CBD. This increase in morphine exposure in our model was also seen in the brain tissue of virtual subjects in both populations. While results from this study show that concomitant use of CBD and morphine will likely not lead to a dramatic pharmacokinetic DDI, dosage adjustments and patient monitoring may be needed to ensure morphine plasma and brain concentrations remain within the narrow therapeutic window to ensure patient safety and efficacy. Future studies should also investigate the potential DDI between CBD and other commonly prescribed opioids either through PBPK modeling or clinical studies.

## Figures and Tables

**Table 1:** PBPK-model-predicted and observed (mean and 95% CI) morphine exposure in healthy and cirrhotic populations with and without CBD administration.

	Sim-Healthy				
	Dose (mg)	AUC (ng·h/mL)	95% CI	C_max_ (ng/mL)	95% CI
**Morphine Solution**					
Morphine	11.7	36.8	34.9–38.9	12.7	12.2–13.3
Morphine + CBD		39.0	37.0–41.2	13.5	13.0–14.1
Obs/Pred Ratio		1.06		1.06	
**Morphine IR**					
Morphine	15.2	48.4	45.8–51.0	16.8	16.0–17.5
Morphine + CBD		51.2	48.5–5430	17.8	17.0–18.6
Obs/Pred Ratio		1.06		1.06	
**Morphine CR**					
Morphine	22.6	70.0	66.3–73.9	11.4	10.9–11.9
Morphine + CBD		72.6	68.8–76.6	11.9	11.4–12.5
Obs/Pred Ratio		1.04		1.05	
	**Sim-Cirrhosis**				
	**Dose (mg)**	**AUC (ng·h/mL)**	**95% CI**	**C_max_ (ng/mL)**	**95% CI**
**Morphine Solution**					
Morphine	11.7	119.1	113.9–124.6	39.4	38.2–40.5
Morphine + CBD		127.9	122.2–133.9	41.7	40.5–43.0
Obs/Pred Ratio		1.07		1.06	
**Morphine IR**					
Morphine	15.2	155.7	148.9–162.8	51.5	50.0–53.0
Morphine + CBD		167.0	159.6–174.9	54.5	52.9–56.1
Obs/Pred Ratio		1.07		1.06	
**Morphine CR**					
Morphine	22.6	223.7	213.9–234.0	37.1	36.0–38.3
Morphine + CBD		236.3	225.8–247.4	39.2	37.9–40.5
Obs/Pred Ratio		1.06		1.06	

Abbreviations: IR—immediate release; CR—controlled release; AUC—area under the plasma concentration–time curve; C_max_—maximum plasma concentration; Obs/Pred Ratio—Observed/Predicted ratio.

## Data Availability

The original contributions presented in the study are included in the article/Supplementary Materials, further inquiries can be directed to the corresponding authors.

## Supplementary Materials

The following supporting information can be downloaded at https://www.mdpi.com/article/10.3390/pharmaceutics16121599/s1, Figure S1: PBPK model of predicted and observed morphine plasma pharmacokinetic profiles after IV administration in healthy subjects. Data points represent the observed mean systemic plasma morphine concentrations (Stuart-Harris et al., 2000; Lotsch et al., 1998; Hoskin et al., 1989; Hasselstrom and Sawe, 1993; Osborne et al., 1990) [6,42,55,56,57] and the green line represents the model-predicted CBD plasma concentration–time profile. Gray lines indicate the 5th–95th percentile of the virtual population. Figure S2: PBPK model of predicted and observed plasma morphine pharmacokinetic profiles after (A) immediate release (Osborne et al., 1990; Masood et al., 1996; Hasselstrom and Sawe, 1993) [6,57,58] and (B) controlled release (Hoskin et al., 1989; Preechagoon et al., 2010; Kaiko et al., 1992; Drake et al., 1996) [37,42,59,60] oral administration healthy populations. Data points represent the observed mean systemic plasma morphine concentrations, and the green line represents the model-predicted CBD plasma concentration–time profile. Gray lines indicate the 5th–95th percentile of the virtual population. Figure S3: PBPK model of predicted and observed morphine CSF and brain concentrations after IV administration of 0.3 mg/kg of morphine in cancer patients (Meineke et al., 2002) [61]. Data points represent the observed values, and the green line represents the model-predicted morphine CSF or total brain concentration–time profiles. Gray lines indicate the 5th–95th percentile of the virtual population. Figure S4: PBPK model of predicted and observed CBD plasma pharmacokinetic profiles after IV administration of 20 mg CBD in healthy adults (Ohlsson et al., 1986) [62]. Data points represent the observed values, and the green line represents the model-predicted CBD plasma concentration–time profile. Gray lines indicate the 5th–95th percentile of the virtual population. Figure S5: PBPK model of predicted and observed (Taylor et al., 2018) [45] CBD plasma pharmacokinetic profiles after oral administration of CBD in healthy adults in the fasted state. Data points represent the observed mean systemic plasma CBD concentrations, and the green line represents the model-predicted CBD plasma concentration–time profile. Gray lines indicate the 5th–95th percentile of the virtual population. Figure S6: Sensitivity analysis for the change of inhibition constant (Ki) of CBD on the ratio of simulated morphine AUC. The horizontal black dashed line indicates the AUCR cutoff of 1.25 and the vertical red dashed line indicates the Ki value determined experimentally. Figure S7: Simulated morphine exposure with (red) and without (blue) CBD in (A) healthy, and (B) hepatic-impaired virtual populations, after oral administration of morphine (15.2 mg IR tablet) and CBD (1500 mg twice daily for 7 days). 95% confidence intervals are shaded. Table S1: Key physiochemical and system specific input parameters for the development of physiologically based pharmacokinetic model for morphine using Simcyp v23. Table S2: Key physiochemical and system specific input parameters for the development of physiologically based pharmacokinetic model for cannabidiol using Simcyp v23. Table S3: PBPK model-predicted and observed (mean and 90% CI) morphine exposure in healthy and cirrhotic adults after single intravenous or oral dose of morphine. Table S4: PBPK model-predicted and observed (mean and 90% CI) CBD exposure in healthy and cirrhotic adults after single intravenous or oral dose of CBD. References [6,22,23,26,29,30,31,32,33,35,36,37,38,40,41,42,43,44,45,55,56,57,58,59,60,61,62,63,64,65,66] are cited in the supplementary materials.
