# Supplementary material for: Evaluation of the Drug–Drug Interaction Potential of Cannabidiol Against UGT2B7-Mediated Morphine Metabolism Using Physiologically Based Pharmacokinetic Modeling
Source: Pharmaceutics. 2024 Dec 16;16(12):1599. doi: 10.3390/pharmaceutics16121599 (PMC11678041; doi:10.3390/pharmaceutics16121599)

Figure 1

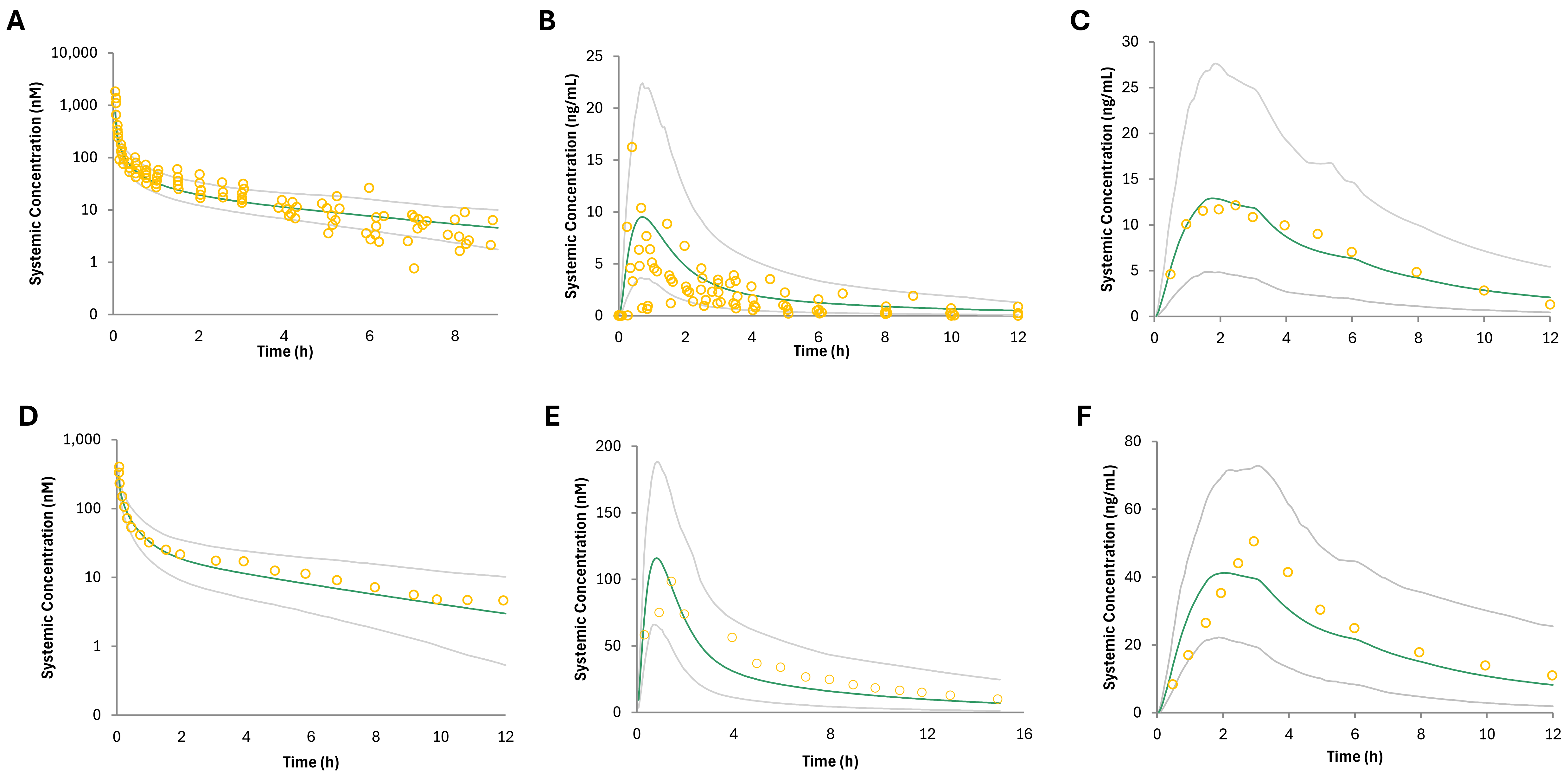
A
Systemic Concentration (nM)
10,000
1,000
100
10
1
0
0
2
4
6
8
Time (h)
B
Systemic Concentration (ng/mL)
25
20
15
10
5
0
0
2
4
6
8
10
12
Time (h)
C
Systemic Concentration (ng/mL)
30
25
20
15
10
5
0
0
2
4
6
8
10
12
D
Systemic Concentration (nM)
1,000
100
10
1
0
0
2
4
6
8
10
12
Time (h)
E
Systemic Concentration (nM)
200
150
100
50
0
0
4
8
12
16
Time (h)
F
Systemic Concentration (ng/mL)
80
60
40
20
0
0
2
4
6
8
10
12
Time (h)

Figure 2

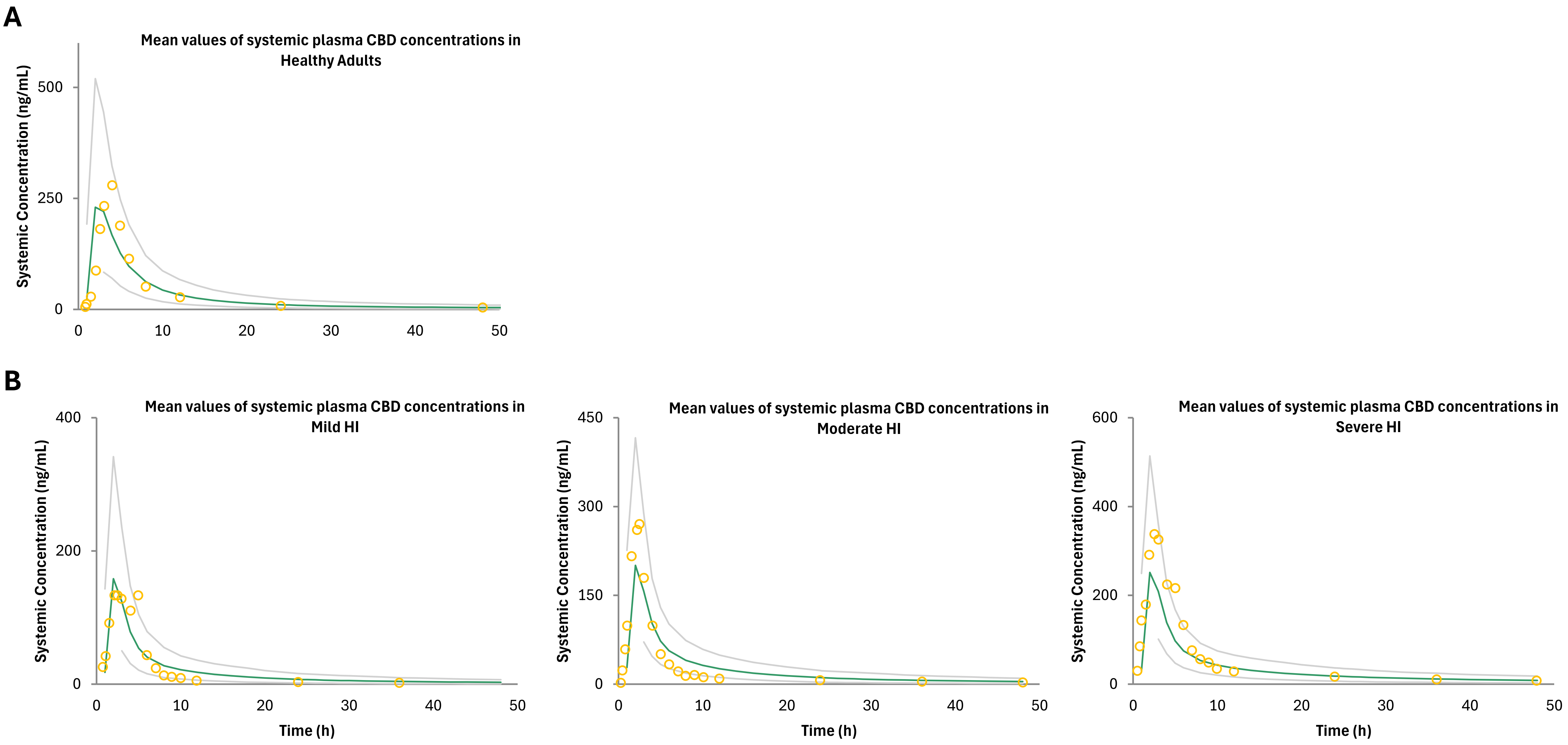
A
Mean values of systemic plasma CBD concentrations in Healthy Adults
Systemic Concentration (ng/mL)
0
250
500
0
10
20
30
40
50
B
Mean values of systemic plasma CBD concentrations in Mild HI
Systemic Concentration (ng/mL)
0
200
400
Time (h)
Mean values of systemic plasma CBD concentrations in Moderate HI
Systemic Concentration (ng/mL)
0
150
300
450
Time (h)
Mean values of systemic plasma CBD concentrations in Severe HI
Systemic Concentration (ng/mL)
0
200
400
600
Time (h)

Figure 3

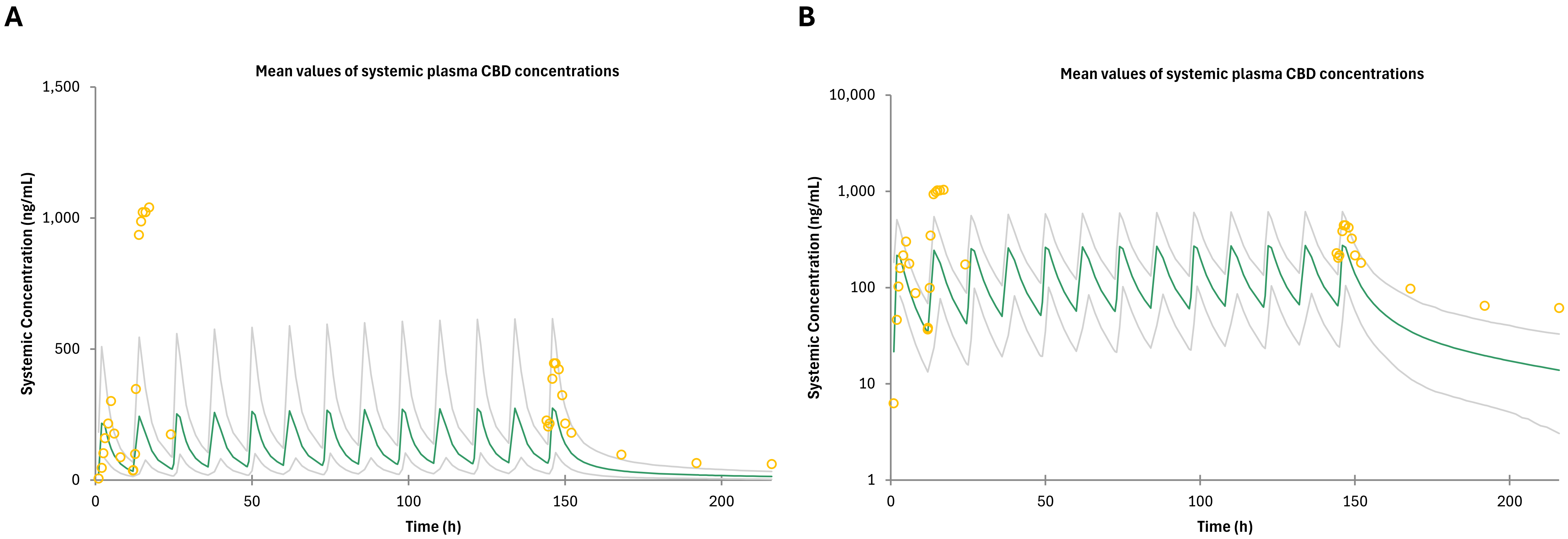
A
Mean values of systemic plasma CBD concentrations
Systemic Concentration (ng/mL)
1,500
1,000
500
0
0
50
100
150
200
Time (h)
B
Mean values of systemic plasma CBD concentrations
Systemic Concentration (ng/mL)
10,000
1,000
100
10
1
0
50
100
150
200
Time (h)

Figure 4

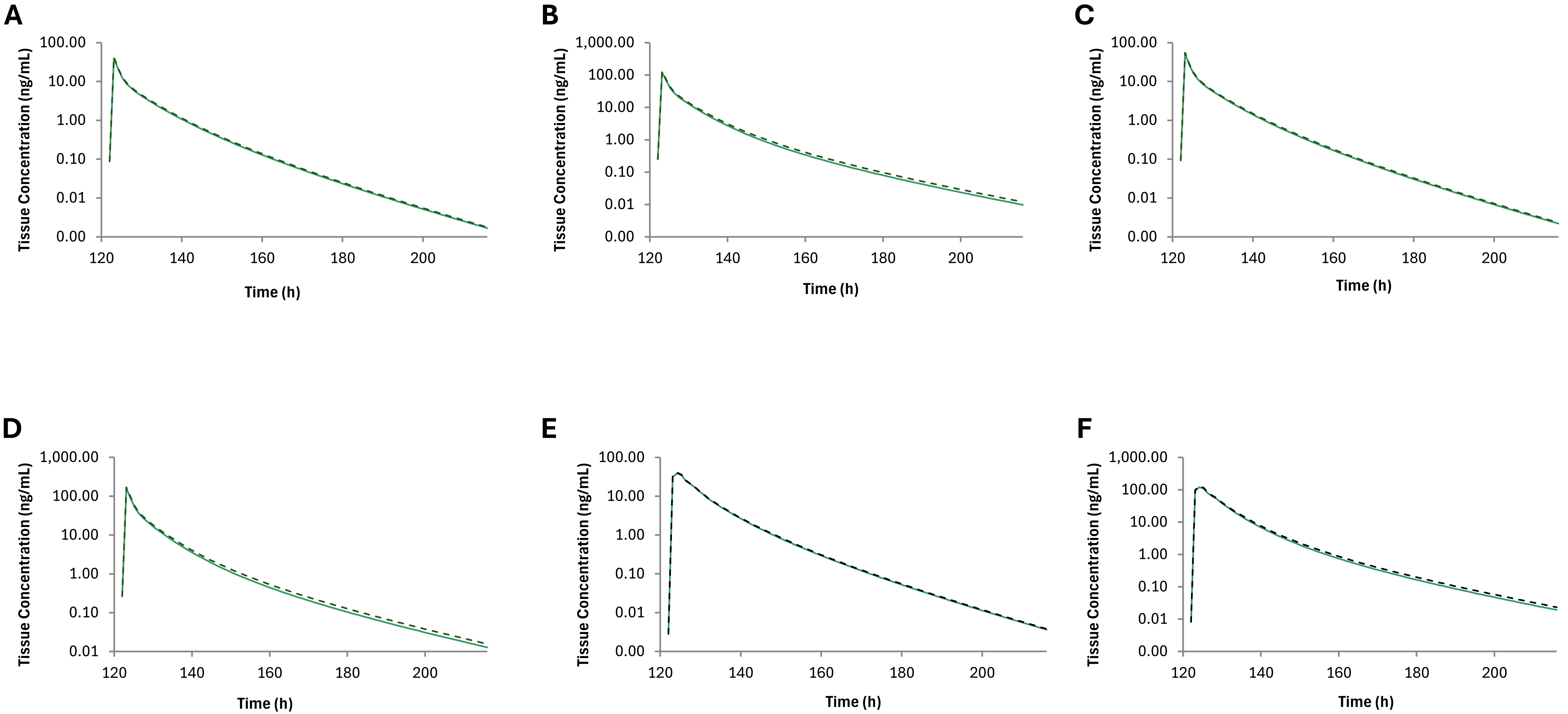
A
Tissue Concentration (ng/mL)
100.00
10.00
1.00
0.10
0.01
0.00
120
140
160
180
200
Time (h)
B
Tissue Concentration (ng/mL)
1,000.00
100.00
10.00
1.00
0.10
0.01
0.00
120
140
160
180
200
Time (h)
C
Tissue Concentration (ng/mL)
100.00
10.00
1.00
0.10
0.01
0.00
120
140
160
180
200
Time (h)
D
Tissue Concentration (ng/mL)
1,000.00
100.00
10.00
1.00
0.10
0.01
120
140
160
180
200
Time (h)
E
Tissue Concentration (ng/mL)
100.00
10.00
1.00
0.10
0.01
0.00
120
140
160
180
200
Time (h)
F
Tissue Concentration (ng/mL)
1,000.00
100.00
10.00
1.00
0.10
0.01
0.00
120
140
160
180
200
Time (h)

Figure 5

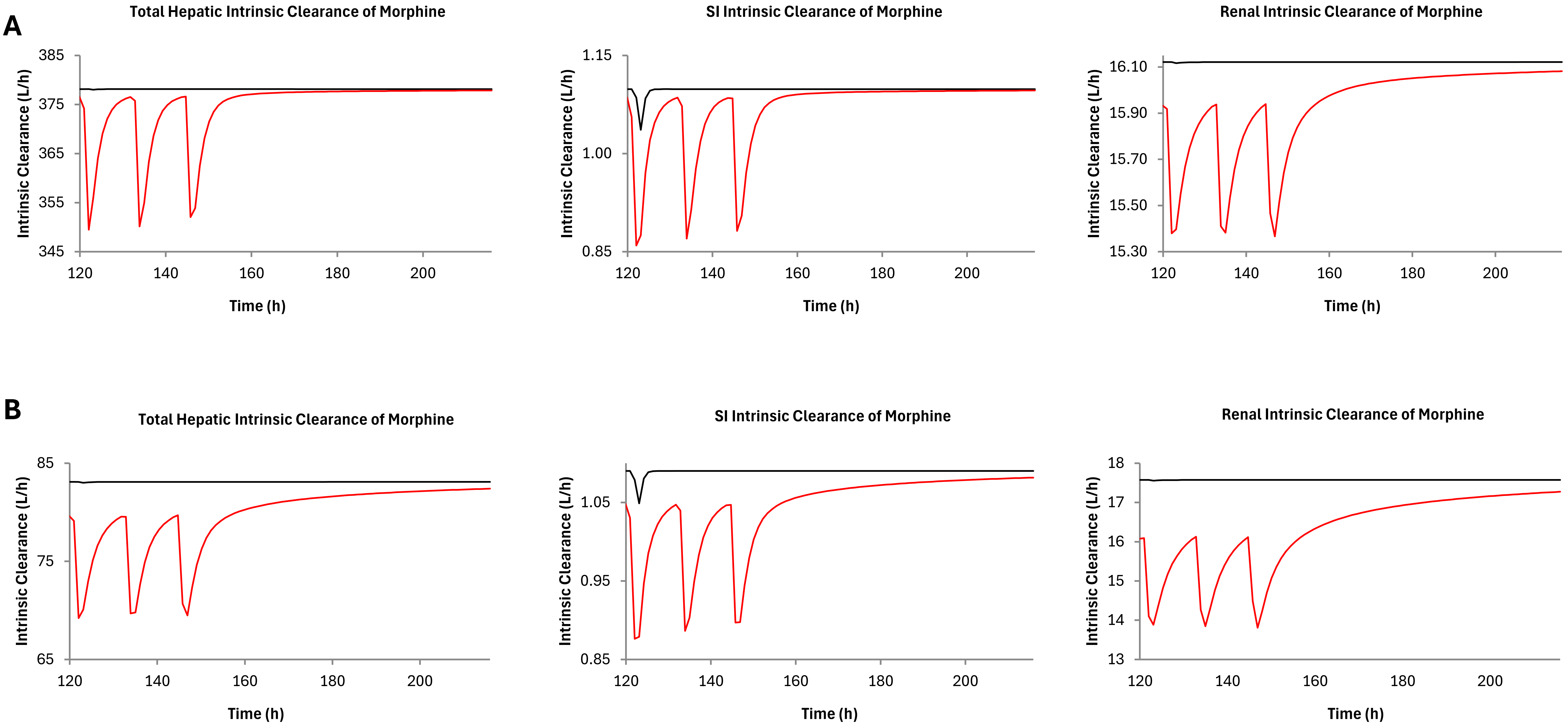
A
Total Hepatic Intrinsic Clearance of Morphine
Intrinsic Clearance (L/h)
385
375
365
355
345
120
140
160
180
200
Time (h)
SI Intrinsic Clearance of Morphine
Intrinsic Clearance (L/h)
1.15
1.00
0.85
120
140
160
180
200
Time (h)
Renal Intrinsic Clearance of Morphine
Intrinsic Clearance (L/h)
16.10
15.90
15.70
15.50
15.30
120
140
160
180
200
Time (h)
B
Total Hepatic Intrinsic Clearance of Morphine
Intrinsic Clearance (L/h)
85
75
65
120
140
160
180
200
Time (h)
SI Intrinsic Clearance of Morphine
Intrinsic Clearance (L/h)
1.05
0.95
0.85
120
140
160
180
200
Time (h)
Renal Intrinsic Clearance of Morphine
Intrinsic Clearance (L/h)
18
17
16
15
14
13
120
140
160
180
200
Time (h)

Supplemental Figure 1

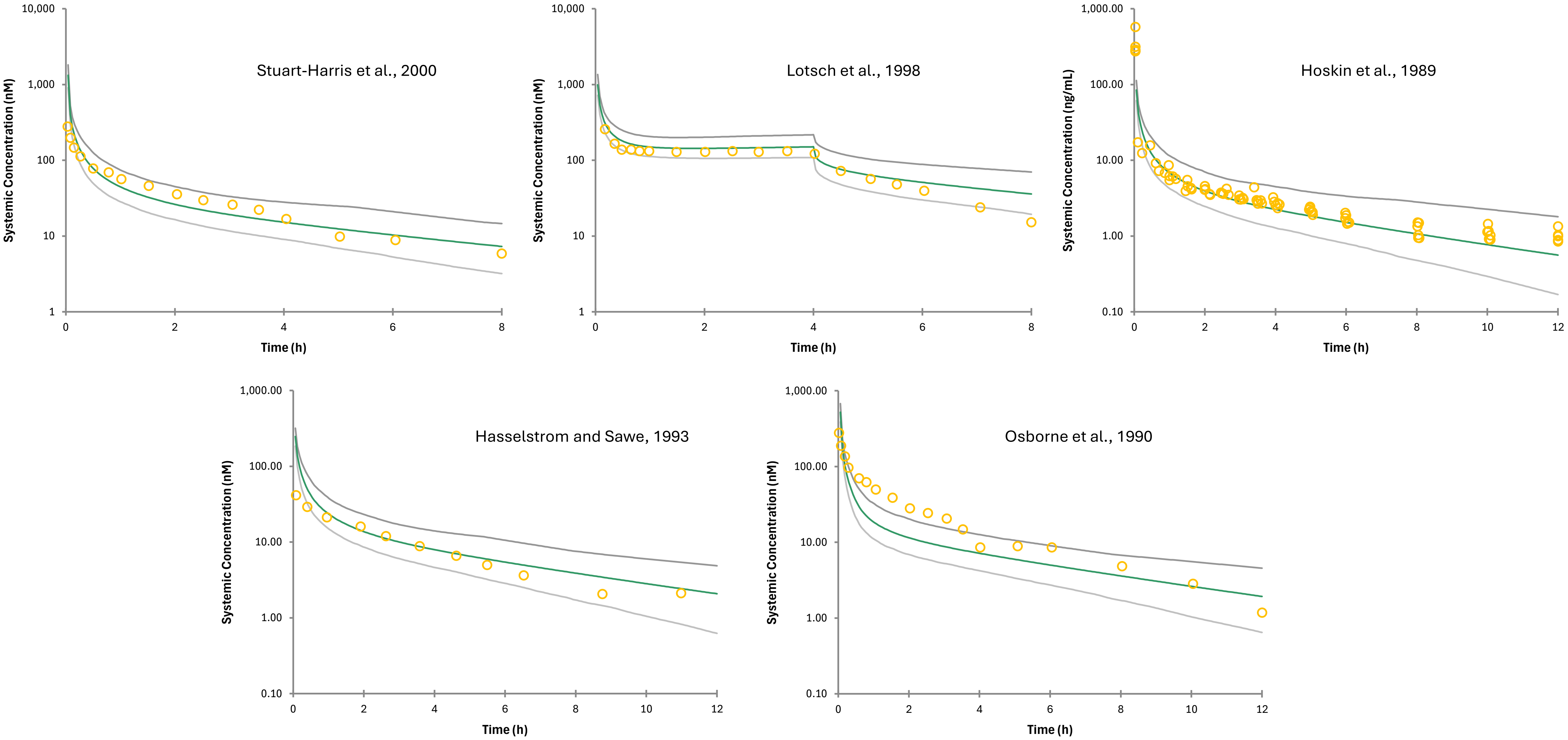

Supplemental Figure 2

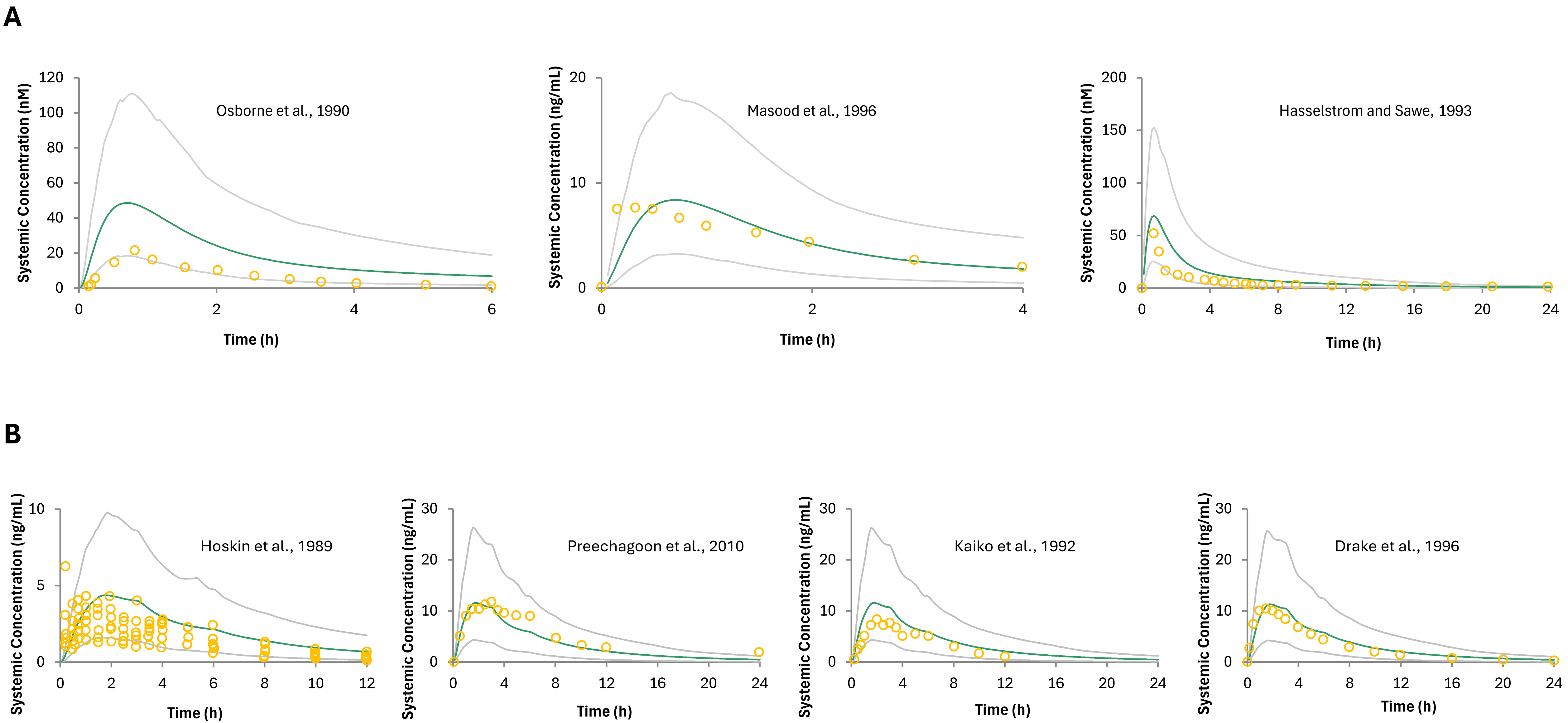

Supplemental Figure 3

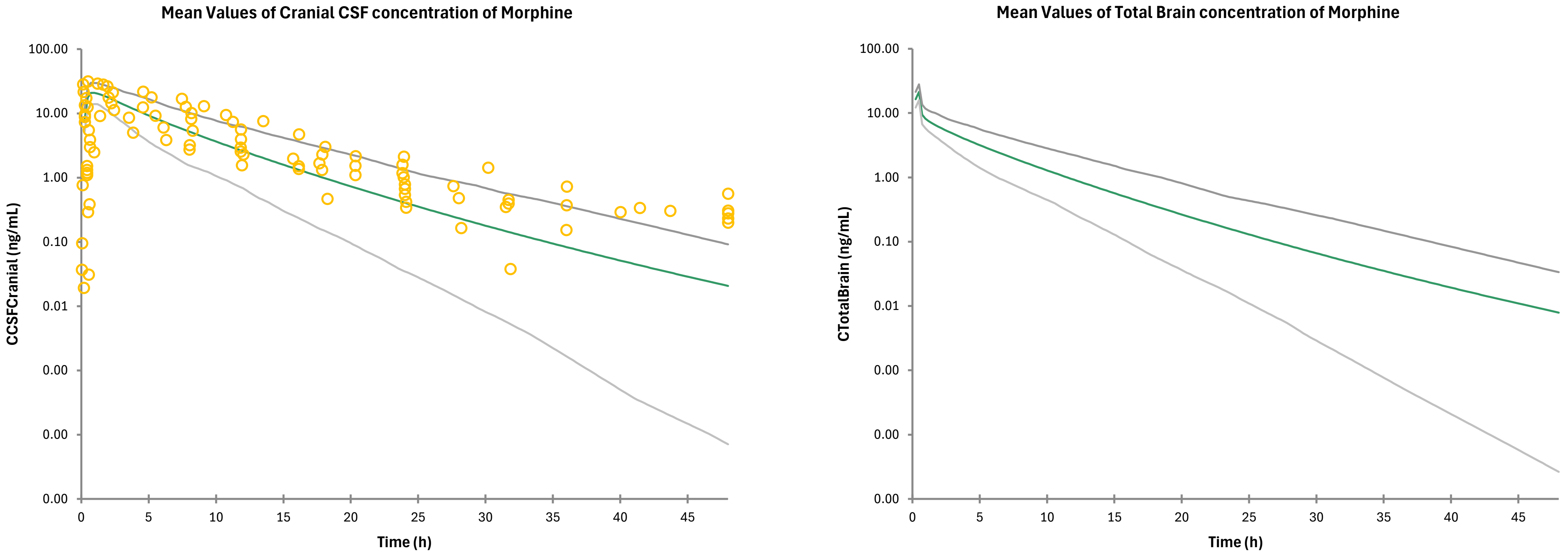

Supplemental Figure 4

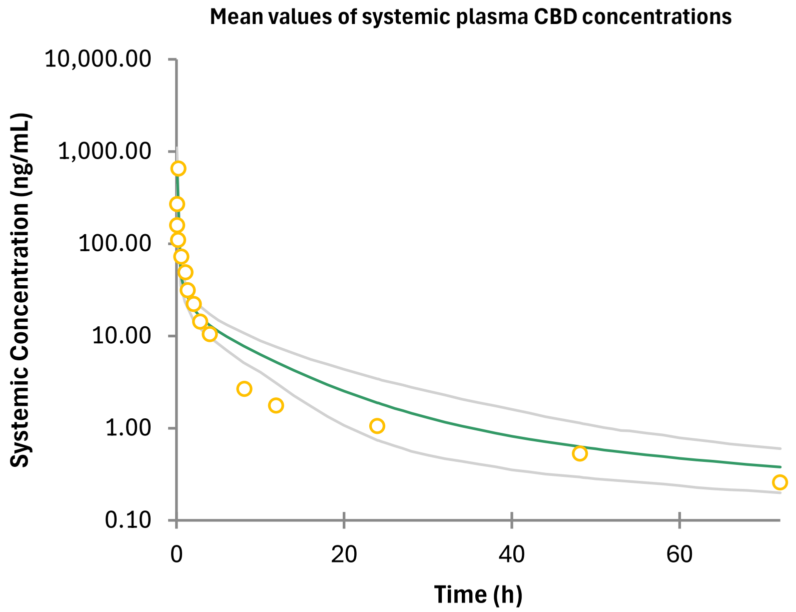

Supplemental Figure 5

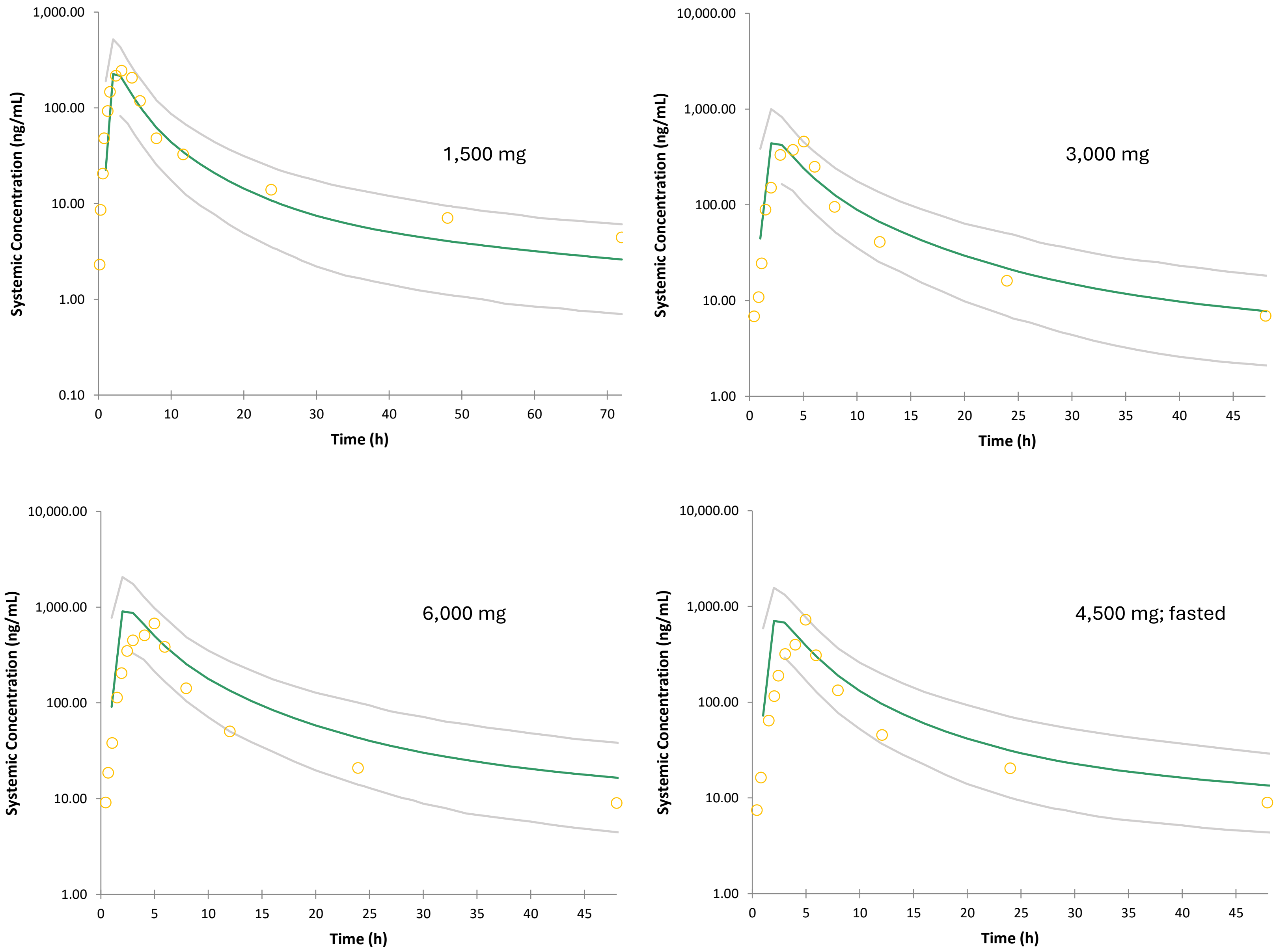

Supplemental Figure 6

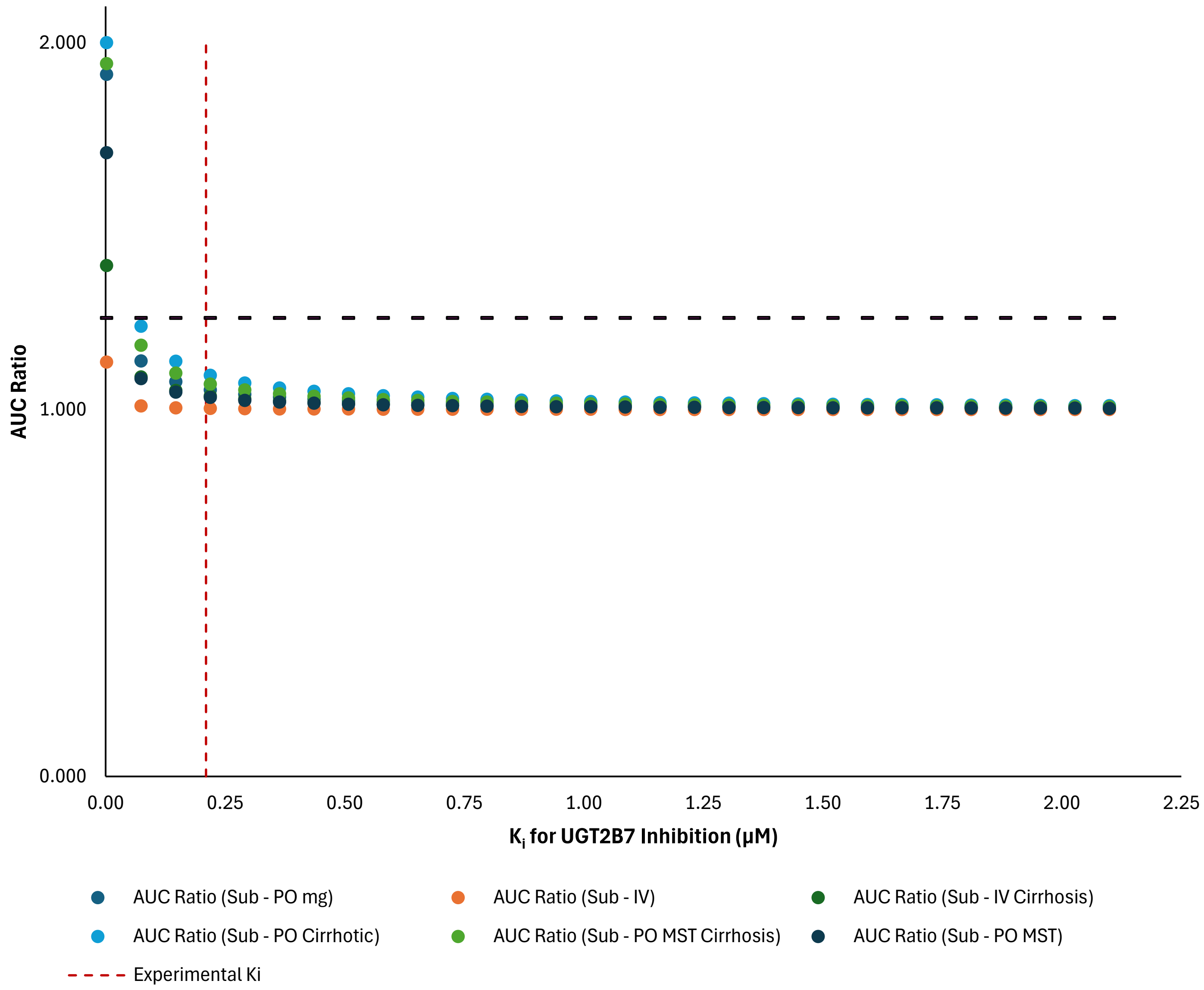

Supplemental Figure 7

A

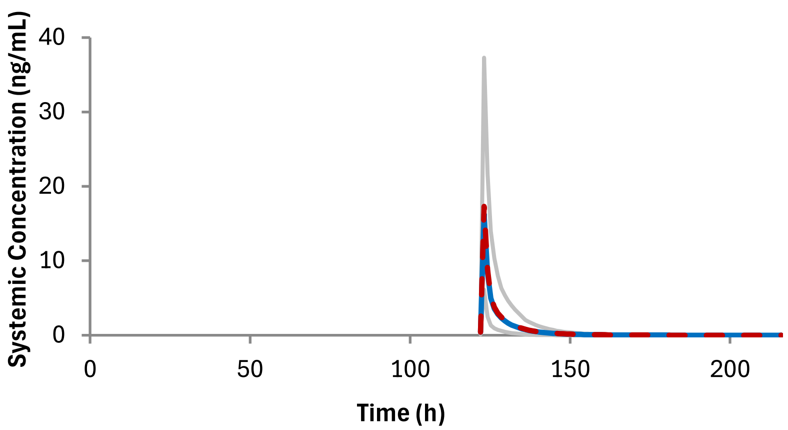

B

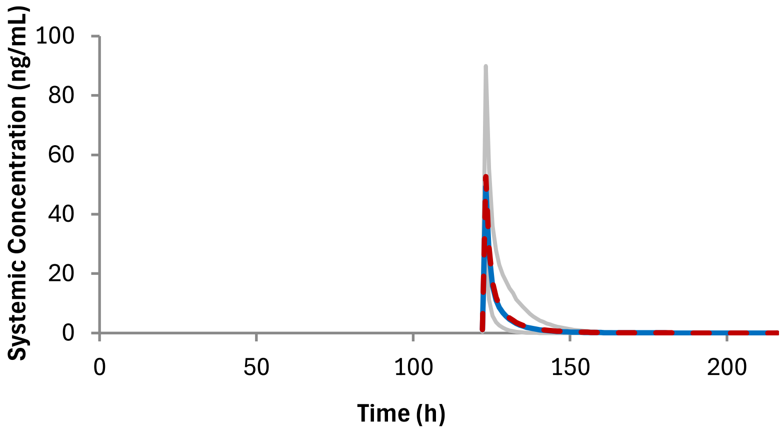

Supplement: Supplementary file 1 [file pharmaceutics-16-01599-s001.zip › Coates et al_Morphine PBPK Modeling Paper_Figures_Dec 11 2024.pdf]
