# Supplementary material for: Evaluation of the Drug–Drug Interaction Potential of Cannabidiol Against UGT2B7-Mediated Morphine Metabolism Using Physiologically Based Pharmacokinetic Modeling
Source: Pharmaceutics. 2024 Dec 16;16(12):1599. doi: 10.3390/pharmaceutics16121599 (PMC11678041; doi:10.3390/pharmaceutics16121599)
Supplement: Supplementary file 1 [file pharmaceutics-16-01599-s001.zip › Coates et al_Morphine PBPK_Supplementary Methods_Nov 3 2024.pdf]

Supplementary Materials

Shelby Coates*, Keti Bardhi, Bhagwat Prasad, Philip Lazarus*+

Department of Pharmaceutical Sciences, College of Pharmacy and Pharmaceutical Sciences, Washington State University, Spokane, WA 99202

***Corresponding authors:**

Shelby Coates, Department of Pharmaceutical Sciences, College of Pharmacy and Pharmaceutical Sciences, Washington State University, 412 E. Spokane Falls Blvd, Spokane, WA 99202. E-mail: Shelby.coates@wsu.edu

Philip Lazarus, Department of Pharmaceutical Sciences, College of Pharmacy and Pharmaceutical Sciences, Washington State University, 412 E. Spokane Falls Blvd, Spokane, WA 99202. E-mail: phil.lazarus@wsu.edu

+ Philip Lazarus, Division of Quantitative Molecular Biosciences, Department of Pharmaceutical Sciences, School of Pharmacy and Pharmaceutical Sciences, SUNY University at Buffalo, 160 Hayes Rd. Buffalo, NY 14215. E-mail: plazarus@buffalo.edu

CBD PBPK Model Development

CBD's clearance, permeability (perfusion-limited), and absorption profile was based upon the model of Bansal et al., [1], utilizing the first-order absorption model. A full PBPK model based upon the Poulin and Theil [2] method was used to predict CBD distribution as described in Bansal et al., [1]. The model was validated in healthy and hepatically impaired populations (mild, moderate, and severe) as described in Bansal et al., [1] utilizing the Simcyp Child-Pugh A-C models and modifications as described above. The expression level of UGT2B7 and UGT1A9 was altered in the Child-Pugh C model to better describe the decrease in expression level seen in cirrhotic adults as compared to healthy adults [1,3].

GMFE and MRD Calculations

Model validation was also statistically analyzed by determining the mean relative deviation (MRD) and geometric mean fold error (GMFE) of both the AUC and C_{max} predicted to observed ratios with acceptable models equal to or below 2 using the following equations:

$$MRD = 10^{\sqrt{\frac{\sum_{i=1}^{k}\left(\log_{10}\hat{c_i} - \log_{10}c_i\right)^2}{k}}} \quad (1)$$

Where c_i = i-th observed plasma concentration, $\hat{c_i}$ = predicted plasma concentration corresponding to the i-th observed plasma concentration, and k = number of observed values.

$$GMFE = 10^{\sqrt{\frac{\sum_{i=1}^{m}\left|\log_{10}\left(\frac{\widehat{p_i}}{p_i}\right)\right|}{m}}} \quad (2)$$

Where p_i = observed AUC_{last} or C_{max} value of study i, $\widehat{p_i}$ = corresponding predicted AUC_{last} or C_{max} value of study i, and m = number of studies.

CBD – morphine DDI workspace in virtual healthy and hepatic impairment populations.

Substrate	
Compound Name	Morphine_PO
Version number	Not applicable
Molecule Type	Small Molecule
Route	Oral
Dose Units	mg
Dose	15.200
Start Day	6.000
Start Time	10h0m
Dosing Regimen	Single Dose
PhysChem and Blood Binding	
Mol Weight (g/mol)	285.343
log P	0.770
Compound Type	Diprotic Base
pKa 1	9.630
pKa 2	7.930
Polar Surface Area (Å²)	52.930
Hydrogen bond donors	2.000
Hydrogen bond acceptors	4.000
Extended Clearance Classification System	Class 2 - Metabolism
BP input type	User
B/P	1.080
Haematocrit	45.000
fu Input	User

Inhibitor 1	
Compound Name	Cannabidiol_PO_Fasted_multiple dose
Version number	Not applicable
Molecule Type	Small Molecule
Route	Oral
Dose Units	mg
Dose	1500.000
Start Day	1.000
Start Time	8h0m
Dosing Regimen	Multiple Dose
Dose Interval (h)	12.000
Number of Doses	13.000
PhysChem and Blood Binding	
Mol Weight (g/mol)	314.500
log P	6.330
Compound Type	Diprotic Acid
pKa 1	9.130
pKa 2	9.130
Polar Surface Area (Å²)	40.460
Hydrogen bond donors	2.000
Hydrogen bond acceptors	2.000
Extended Clearance Classification System	Class 1A - Metabolism
BP input type	User
B/P	0.670

fu	0.620
Reference Binding Component	HSA
Protein Reference Conc (g/L)	45.000
% Bound to Lipoprotein	0.000
% Bound to Lipoprotein (CV %)	0.000
Absorption	
Absorption Model	ADAM
Use UBL fluid volumes	No
fu(Gut) input type	User
fu(Gut)	1.000
Peff,man Type	Global
Peff,man (10-4 cm/s)	2.070
Luminal Degradation	Discrete
Degradation Rate Stomach (1/h)	0.000
Degradation Rate Duodenum (1/h)	0.000
Degradation Rate Jejunum I (1/h)	0.000
Degradation Rate Jejunum II (1/h)	0.000
Degradation Rate Ileum I (1/h)	0.000
Degradation Rate Ileum II (1/h)	0.000
Degradation Rate Ileum III (1/h)	0.000
Degradation Rate Ileum IV (1/h)	0.000
Degradation Rate Colon (1/h)	0.000
Input Form	Solid
Dual Solid State Formulation	No
DLM Particle Handling Model	Particle Population Balance

Haematocrit	45.000
fu Input	User
fu	0.013
Reference Binding Component	HSA
Protein Reference Conc (g/L)	45.000
% Bound to Lipoprotein	0.000
% Bound to Lipoprotein (CV %)	0.000
Absorption	
Absorption Model	1st order
Input type	User
fa	0.250
CV fa (%)	30.000
ka (1/h)	0.550
CV ka (%)	30.000
lag time (h)	1.500
CV lag time (%)	30.000
fu(Gut) input type	User
fu(Gut)	0.013
Q(Gut) input type	User
Q(Gut) (L/h)	2.500
CV Q(Gut) (%)	30.000
Peff,man Type	n/a
Permeability Method	Mechanistic Model
Effective Concentration for Permeation	Free Aqueous
UBL Diffusion Coefficient Scalar	1.000

Formulation	IR: DLM Model
Define Disintegration Profile	Not activated
Salt Form	No
Dissolution Type	Solubility
Solubility Type	Intrinsic (predicted)
Interpolation Method	Linear
Solubility (mg/mL)	0.768
So_scalar	Global
Global So_scalar	1.000
Surface Solubility Option	Bulk Fluid Solubility
Supersaturation Precipitation Model	First Order
FO Precipitation Model	Model 2
PRC (Precipitation Rate Constant)	Global
PRC (1/h)	4.000
CSR (Critical Supersaturation Ratio)	Global
CSR value	10.000
Reference Concentration for Precipitation Model	Total
Salt Limited Solubility Model	Ksp Model
Counterion Id	Counterion 1
Drug Solubility at pHmax Option	User Input
Drug Solubility at pHmax (mg/mL)	10.000
Ksp Option	Predicted
Ksp Value (mM2 or mM3)	823.020
Drug : Counterion Stoichiometry	One : One
Counterion Type	Monoprotic Strong Acid
Counterion	Hydrochloric acid (Cl-)
Concentration of counterion in drink (mg/mL)	0.000

Intrinsic Transcellular Permeability input type	Predicted
Prediction method	logPo:w Method2 (for acids)
logPo:w	6.330
C-efficient, a	2.320
C-efficient, b	0.427
C-efficient, c	-5.172
C-efficient, d	-0.464
C-efficient, e	0.234
Ptrans,0 Cap input type	Default
Include Ion Transcellular Permeation	No
Apply Accessible Surface Area Scalar	Yes
Force Unstirred boundary layer pH to bulk pH	No
Paracellular Effective Molecular Radius Method	Predicted
Paracellular Scalar	0.00
Pore Electric Gradient Potential Drop (mV)	-64.145
Distribution	
Distribution Model	Full PBPK Model
Replacement Organ?	No
Organ Replaced	n/a
User-defined Additional Organ	No
Type	n/a
Vss input type	Predicted
Prediction Method	Method 1
Concentration-dependent volume	No
log Po:w	6.330

Endogenous Ion	Yes
Counterion Molecular Weight (g/mol)	36.460
Counterion compound pKa1	-6.000
Dispersion Type	Monodispersed
Input Type	Volume Fraction
MonoDispersed Radius (µm)	10.000
No. of Bins (Simulation)	30.000
Minimum Radius (µm)(Simulation)	0.100
Maximum Radius (µm)(Simulation)	11.000
Particle Drift Effect model	Off
Step Type(Simulation)	Uniform
DLM Scalar	All Segments
DLM Scalar values	1.000
Particle density (g/mL)	1.200
Viscosity Model	Off
Viscosity Model Exponent	0.987
Diffusion coeff. input type	Predicted
Diffusion coeff value Uncharged (1 cP, 37°C) (10-4 cm²/min)	4.816
Diffusion coeff value Ion (1 cP, 37°C) (10-4 cm²/min)	4.816
Diffusion coeff, micelle (1 cP, 37°C) (10-4 cm²/min) mean	0.780
Diffusion coeff, micelle CV (%)	20.000
heff method selected	Hintz-Johnson
heff cut-off type	Default
heff cut-off value (µm)	30.000
Bile Micelle mediated solubilization	On
Bile solubilization input type	Predicted
Concentration-dependent logKm:w	Off

fut input type	Predicted
logDvo:w (pH=7.4) input type	Predicted
logP vo:w input type	Predicted
logP vo:w Prediction Method	Hansch
logP vo:w Hansch a	1.115
logP vo:w Hansch b	-1.350
Compound Type	Diprotic Acid
pKa 1	9.130
pKa 2	9.130
Adipose input type	Predicted
Bone input type	Predicted
Brain input type	Predicted
Gut input type	Predicted
Heart input type	Predicted
Kidney input type	Predicted
Liver input type	Predicted
Lung input type	Predicted
Muscle input type	Predicted
Skin input type	Predicted
Spleen input type	Predicted
Pancreas input type	Predicted
Kp Scalar	1.000
Smoothing Function	Off
Lipid Binding Scalar	1.000
Use Pvo:w for Neutral Lipid Partition in All Tissues	No
Elimination	

Bile Micelle Partition: Slope	0.740
Bile Micelle Partition: Offset	2.290
Bile Micelle Partition: Ionised Species Correction	2.000
Absorption Scalars (no units)	SI Global
Absorption Scalar SI Global	1.000
Absorption Scalar Colon	1.000
Portal Vein Uptake Driving Concentration	Total
Basolateral Permeability Scalars (no units)	SI Global
Basolateral Permeability Scalar SI Global	0.100
Basolateral Permeability Scalar Colon	1.000
Segregated transit time model	Activated
Use only ascending colon transit time	Not activated
MRT of particles and pellets may be shorter than fluid	Not activated
Fluid stomach lag time (h) (mean)	0.000
Fluid stomach lag time (%CV)	0.000
Fluid stomach post lag MRT (h) (mean)	0.270
Fluid stomach post lag MRT (%CV)	36.000
Fluid small intestinal MRT (h) (mean)	3.400
Fluid small intestinal MRT (%CV)	9.190
Fluid colon MRT (male) (h) (mean)	37.500
Fluid colon MRT (male) (h) (%CV)	32.000
Fluid colon MRT (female) (h) (mean)	55.750
Fluid colon MRT (female) (h) (%CV)	32.000
Particle stomach lag time (h) (mean)	0.000
Particle stomach lag time (%CV)	0.000
Particle stomach post lag MRT (h) (mean)	0.270
Particle stomach post lag MRT (%CV)	36.000

Clearance Type	Enzyme Kinetics
FI Correction	Not used
PLR Correction	Not Used
In vitro metabolic system	HLM
Pathway	Pathway 1
Enzyme	CYP3A4
CLint (µL/min/mg protein)	220.000
fu mic	1.000
Pathway	Pathway 1
Enzyme	CYP1A2
CLint (µL/min/mg protein)	56.000
fu mic	1.000
Pathway	Pathway 1
Enzyme	CYP2B6
Genotype	*1/*1
CLint (µL/min/mg protein)	46.000
fu mic	1.000
Pathway	Pathway 1
Enzyme	CYP2C8
CLint (µL/min/mg protein)	51.000
fu mic	1.000

Particle small intestinal MRT (h) (mean)	3.400
Particle small intestinal MRT (%CV)	9.190
Particle colon MRT (male) (h) (mean)	37.110
Particle colon MRT (male) (h) (%CV)	47.000
Particle colon MRT (female) (h) (mean)	52.870
Particle colon MRT (female) (h) (%CV)	47.000
Distribution	
Distribution Model	Full PBPK Model
Replacement Organ?	No
Organ Replaced	n/a
User-defined Additional Organ	No
Type	n/a
Vss input type	Predicted
Prediction Method	Method 2
Concentration-dependent volume	No
log Po:w	0.770
Ka_{AP} input type	Predicted
logP vo:w input type	Predicted
logP vo:w Prediction Method	Hansch
logP vo:w Hansch a	1.115
logP vo:w Hansch b	-1.350
Compound Type	Diprotic Base
pKa 1	9.630
pKa 2	7.930
Adipose input type	Predicted

Pathway	Pathway 1
Enzyme	CYP2C9
Genotype	*1/*1
CLint (µL/min/mg protein)	93.000
fu mic	1.000
Pathway	Pathway 1
Enzyme	CYP2C19
CLint (µL/min/mg protein)	193.000
fu mic	1.000
Pathway	Pathway 1
Enzyme	CYP2D6
CLint (µL/min/mg protein)	38.000
fu mic	1.000
In vitro metabolic system	HLM
Pathway	Pathway 1
Enzyme	UGT1A9
CLint (µL/min/mg protein)	541.000
fu mic	1.000
Pathway	Pathway 1
Enzyme	UGT2B7
CLint (µL/min/mg protein)	2162.000
fu mic	1.000

Bone input type	Predicted
Brain input type	Predicted
Gut input type	Predicted
Heart input type	Predicted
Kidney input type	Predicted
Liver input type	Predicted
Lung input type	Predicted
Muscle input type	Predicted
Skin input type	Predicted
Spleen input type	Predicted
Pancreas input type	Predicted
Kp Scalar	1.000
Smoothing Function	On
Lipid Binding Scalar	1.000
Use Pvo:w for Neutral Lipid Partition in All Tissues	No
Elimination	
Clearance Type	Enzyme Kinetics
Fl Correction	Not used
PLR Correction	Not Used
In vitro metabolic system	HLM
Pathway	Pathway 1
Enzyme	UGT2B7
Vmax (pmol/min/mg protein)	9250.000
Km (µM)	115.800

Feed formed renal metabolite into kidney	No
Use Allelic variants for Enzyme 1	No
Enzyme	CYP2C9
Use Allelic variants for Enzyme 2	No
Enzyme	CYP2B6
Ontogeny Profile - Liver	No Profile Used
Ontogeny Profile - Intestine	No Profile Used
Ontogeny Profile - Kidney	No Profile Used
Biliary CLint (Hep) (µL/min/10^6)	0.000
CV Biliary CLint (Hep) (%)	30.000
Ontogeny Profile	No Profile Used
Active enterohepatic recirculation for IV dose	No
Active Hepatic Scalar (Net)	1.000
CL R (L/h)	0.000
CYPs and/or UGTs Interaction	
Enzyme	CYP1A2
Ki (µM)	0.020
fu mic	1.000
MBI Kapp (µM)	1000000.000
MBI Kinact (1/h)	4.200
MBI fu mic	1.000

fu mic	1.000
Forms Metabolite	Sub Pri Met1
Pathway	Pathway 2
Enzyme	UGT2B7
Vmax (pmol/min/mg protein)	1917.000
Km (µM)	115.800
fu mic	1.000
Forms Metabolite	Sub Pri Met2
Feed formed renal metabolite into kidney	No
Use Allelic variants for Enzyme 1	No
Enzyme	CYP2C9
Use Allelic variants for Enzyme 2	No
Enzyme	CYP2B6
Gut Lumen metabolism	in-active
Additional HLM CLint (µL/min/mg protein)	5.760
Additional HLM CV (%)	30.000
Additional HLM fumic	1.000
Ontogeny Profile - Liver	No Profile Used
Ontogeny Profile - Intestine	No Profile Used
Ontogeny Profile - Kidney	No Profile Used
Biliary CLint (Hep) (µL/min/10^6)	0.000

MIA (pmol/mg microsomal protein)	112.892
Enzyme	CYP2C19
Ki (µM)	0.730
fu mic	1.000
MBI Kapp (µM)	1000000.000
MBI Kinact (1/h)	2.400
MBI fu mic	1.000
MIA (pmol/mg microsomal protein)	8.694
Enzyme	CYP3A4
Ki (µM)	0.106
fu mic	1.000
MBI Kapp (µM)	1000000.000
MBI Kinact (1/h)	4.700
MBI fu mic	1.000
MIA (pmol/mg microsomal protein)	251.121
Enzyme	UGT2B7
Ki (µM)	0.210
fu mic	1.000
MIA (pmol/mg microsomal protein)	120.359
Consider Competitive Inhibition Protection Effect on MBI	No
Co-administration of Multiple Inducers/Suppressors	Default
Co-administration of Multiple UGT Inducers/Suppressors	Default

CV Biliary CLint (Hep) (%)	30.000
Ontogeny Profile	No Profile Used
Percentage available for re-absorption (%)	100.000
Active enterohepatic recirculation for IV dose	No
Active Hepatic Scalar (Net)	1.000
CL R (L/h)	1.840
Transport	
Organ/Tissue	Liver
Model	PerL
CL_{PD}(mL/min/10^6 cells)	0.003
fuIW Type	Predicted
fuEW Type	Predicted
Drug concentration for passive permeability	Unbound (ionized and unionized)
Organ/Tissue	Liver
Transporter	SLC22A1 (OCT1)
Location	Sinusoidal
Function	Influx
Jmax (pmol/min/million cells)	29.000
Km (µM)	3.400

Transport	
Organ/Tissue	Tumor
Tumor Permeability limited Model	Off

fuinc	1.000		
System	User		
RAF/REF	5.100		
Organ/Tissue	Tumour		
Tumor Permeability limited Model	Off		

References

1. Bansal, S.; Ladumor, M.K.; Paine, M.F.; Unadkat, J.D. A Physiologically-Based Pharmacokinetic Model for Cannabidiol in Healthy Adults, Hepatically-Impaired Adults, and Children. *Drug Metabolism and Disposition* **2023**, *51*, 743-752, doi:10.1124/dmd.122.001128.
2. Poulin, P.; Theil, F.P. Prediction of pharmacokinetics prior to in vivo studies. 1. Mechanism-based prediction of volume of distribution. *J Pharm Sci* **2002**, *91*, 129-156, doi:10.1002/jps.10005.
3. El-Khateeb, E.; Achour, B.; Al-Majdoub, Z.M.; Barber, J.; Rostami-Hodjegan, A. Non-uniformity of Changes in Drug-Metabolizing Enzymes and Transporters in Liver Cirrhosis: Implications for Drug Dosage Adjustment. *Molecular Pharmaceutics* **2021**, *18*, 3563-3577, doi:10.1021/acs.molpharmaceut.1c00462.
