# Supplementary material for: Evaluation of the Drug–Drug Interaction Potential of Cannabidiol Against UGT2B7-Mediated Morphine Metabolism Using Physiologically Based Pharmacokinetic Modeling
Source: Pharmaceutics. 2024 Dec 16;16(12):1599. doi: 10.3390/pharmaceutics16121599 (PMC11678041; doi:10.3390/pharmaceutics16121599)
Supplement: Supplementary file 1 [file pharmaceutics-16-01599-s001.zip › Coates et al_Morphine PBPK_Supplementary Tables_11Dec 2024.pdf]

Supplemental Table 1. Key physiochemical and system specific input parameters for the development of physiologically based pharmacokinetic model for morphine using Simcyp v23.

Parameters	Value
**Physiochemical Properties**[a]	
Molecular weight (g/mol)	285.3
Log P	0.77
pK_a 1	9.63
pK_a 2	7.93
**Blood binding**[b]	
Blood to plasma ratio	1.08
Fraction unbound in plasma	0.62
**Absorption**	
ADAM model	
f_a[c]	0.92
k_a[c] (1/hr)	0.90
fu_{gut}	1
Q_{gut}[c] (L/hr)	10.2
Permeability	
P_{eff} man[d] (10^{-4} cm/s)	2.07
**Distribution: Full PBPK model**	
Vss[e] (L/Kg)	3.66
**Elimination**	
Clearance type: Enzyme Kinetic (HLM)[f]	
M3G pathway/ Enzyme UGT2B7	
K_m (µM)	115.8
V_{max} (pmol/min/mg of protein)	9250
M6G pathway/ Enzyme UGT2B7	
K_m (µM)	115.8
V_{max} (pmol/min/mg of protein)	1917
Additional clearance	
CL_R (L/hr)[g]	9.21 for IV route<br>1.84 for oral route
Transport: Permeability limited liver model (PerL)	
Transporter Kinetics[h]	
OCT1 K_m (µM)	3.4
OCT1 J_{max} (pmol/min/mg of protein)	29
**Brain Model**	
BBB	
PSB (L/h)[i]	35
$f_{u,br}$[j]	1
$CL_{ABCB1,vitro}$ (µL/min/pmol)[k]	0.14
Blood-cranial CSF barrier	
PSC (L/h)[i,l]	17.5
$f_{u,csf}$[j]	1
Brain-cranial CSF barrier	
PSE (L/h)[m]	300

Abbreviations: ADAM, advanced dissolution, absorption and metabolism; CL_{int}, intrinsic clearance; CL_R, renal clearance; f_a, fraction absorbed from dosage form; fu_{gut}, fraction unbound in the gut; HLM, human liver microsomes; k_a, first-order absorption rate constant; PBPK, physiologically based pharmacokinetic; K_m, Michaelis-Menten constant; V_{max}, maximum rate of metabolism; J_{max}, maximal flux value; OCT1, organic cation transporter-1; P_{eff}, man, effective permeability in man; pK_a, acid dissociation constant; Log P, log of the partition coefficient of a solute between octanol and water; Q_{gut}, gut blood flow; M3G, morphine-3-glucuronide; M6G, morphine-6-glucuronide; Vss, volume of distribution at steady state; BBB, blood brain barrier; PSB, passive permeability-surface area product on the BBB; PSC, passive

permeability-surface area product on the blood-CSF barrier; PSE, passive permeability-surface area product on the brain-CSF barrier; CSF; Cerebrospinal fluid; $f_{u,br}$; fraction unbound in the brain; $f_{u,csf}$; fraction unbound in the CSF.

[a] Physiochemical data were obtained from the ChEMBL database (https://www.ebi.ac.uk/) [1].

[b] Emoto et al., 2017 [2].

[c] Simcyp predicted.

[d] Simcyp predicted based on the equation Log (P_{eff},man) = 4-2.546-0.011PSA-0.27HBD, where PSA is polar surface area and HBD is hydrogen bond donors.

[e] Simcyp predicted by using Method 2 published by Rodgers et al., 2005 and 2006 [3,4].

[f] Optimized enzyme kinetic parameters were obtained from Krekels et al., 2012 [5].

[g] CL_R was calculated based on fe, fraction excreted unchanged in urine (0.08 and 0.01 for IV and oral routes) (Osborne et al., 1990) [6].

[h] Data obtained from Tzvetkov et al., 2013 [7].

[i] Optimized to recapitulate observed values.

[j] Assumed to be 1.

[k] Verscheijden et al., 2021 [8].

[l] Assumed to be half of PSB.

[m] Assigned given the high permeability of this barrier.

Supplemental Table 2. Key physiochemical and system specific input parameters for the development of physiologically based pharmacokinetic model for cannabidiol using Simcyp v23.

Parameters	Value
**Physiochemical Properties**[a]	
Molecular weight (g/mol)	314.5
Log P	6.33
pK_a	9.13
**Blood binding**	
Blood to plasma ratio[b]	0.67
Fraction unbound in plasma[c]	0.013
**Absorption**[c]	
First-order absorption model	
f_a	0.25[1], 0.5[2]
k_a (1/hr)	0.55[1], 1[2]
fu_{gut}	0.013
Q_{gut} (L/hr)	10.24
Lag time (hr)	1.5[1,2]
**Distribution: Full PBPK model**	
Vss[d] (L/Kg)	16.64
**Elimination**	
Clearance type: Enzyme Kinetic (HLM)[e]	
$CL_{int,\ UGT}$ (µL/min/mg of protein)	
UGT1A9	541
UGT2B7	2162
$CL_{int,\ CYP}$ (µL/min/mg of protein)	
CYP1A2	56
CYP2B6	46
CYP2C8	51
CYP2C9	93
CYP2C19	193
CYP2D6	38
CYP3A4	220
Additional clearance	
CL_R (L/hr)[f]	0
Auto-inhibition[g]	
CYP1A2 inactivation	
$K_{I,u}$ (µM)	0.020
K_{inact} (1/h)	4.2
CYP2C19 inactivation	
$K_{I,u}$ (µM)	0.073
K_{inact} (1/h)	2.4
CYP3A inactivation	
$K_{I,u}$ (µM)	0.106
K_{inact} (1/h)	4.7

Abbreviations: CL_{int}, intrinsic clearance; CL_R, renal clearance; f_a, fraction absorbed from dosage form; fu_{gut}, fraction unbound in the gut; HLM, human liver microsomes; k_a, first-order absorption rate constant; $K_{I,u}$, binding-corrected half-maximal inactivation concentration; PBPK, physiologically based pharmacokinetic; pK_a, dissociation constant; Log P, log of the partition coefficient of a solute between octanol and water; Q_{gut}, gut blood flow; Vss, volume of distribution at steady state.

[a] Physiochemical data were obtained from the ChEMBL database (https://www.ebi.ac.uk/) [1].
[b] Samara et al., 1988 [9].
[c] Bansal et al., 2022 [10].
[d] Simcyp predicted.
[e] Bansal et al., 2023 [11].
[f] Tayo et al., 2020 [12].

[g] Bansal et al., 2020 [13].
[1] Fasted.
[2] Fed.

Supplemental Table 3. PBPK model-predicted and observed (mean and 90% CI) morphine exposure in healthy and cirrhotic adults after single intravenous or oral dose of morphine.

References	Population	Age (range)	# females	Population Size	Dose[a] (mg) & Route	Parameters	Obs	Pred	Pred/Obs
Osborne et al., 1990 [6]	Healthy	25-44	0.3	10 subjects x 40 trials	3.76 IV	AUC (nM•h)	115	132	1.25
Hoskin et al., 1989 [14]	Healthy	26-40	0.7	6 subjects x 68 trials	3.76 IV	AUC (ng•h/mL)	45.9	47.0	1.02
Hasselstrom and Sawe, 1993 [15]	Healthy	27-55	0.6	7 subjects x 58 trials	3.76 IV	AUC (nM•h)	165	170	1.03
Stuart-Harris et al., 2000 [16]	Healthy	20-40	0.5	6 subjects x 67 trials	7.52 IV	AUC (nM•h)	290	295	1.02
Lötsch et al., 1998 [17]	Healthy	24-32	0	20 subjects x 20 trials	Bolus 0.14 mg/kg plus infusion of 0.05 mg/kg for 4 hr	CL (L/hr)[b]	137	88	0.64
Lötsch et al., 2002 [18]	Healthy	23-30	0.5	8 subjects x 50 trials	5.64	AUC (nM•h)	225	223	0.99
**Average GMFEs (range)**						AUC	**1.71 (1.16 – 2.76)**		
**Average MRDs (range)**						AUC	**1.65 (1.01 – 1.56)**		
Hasselstrom et al., 1990 [19]	Cirrhosis	52-72	0.14	8 subjects x 50 trials	3.04 IV	AUC (nM•h)	218	193	0.89
**GMFE**						AUC	**1.68**		
**MRD**						AUC	**1.12**		
Hoskin et al., 1989 [14]	Healthy	26-40	0.7	6 subjects x 67 trials	7.52 oral solution	AUC (ng•h/mL)	22.2	22.6	1.01
						C_{max} (ng/mL)	10.6	8.45	0.80
Osborne et al., 1990 [6]	Healthy	25-40	0.3	10 subjects x 40 trials	11.7 IR tablet	AUC (nM•h)	50.2	97.4	1.93
						C_{max} (nM)	24.5	43.2	1.76
Hasselstrom and Sawe, 1993 [15]	Healthy	27-40	0.6	7 subjects x 58 trials	15.2 IR tablet	AUC (nM•h)	202	173	0.86
						C_{max} (nM)	51.0	61.1	1.20

Masood and Thomas, 1996 [20]	Healthy	22-35	0.2	10 subjects x 40 trials	7.5 oral solution	AUC (nM•h)	21.0	20.3	0.97
						C_{max} (nM)	9.3	7.5	0.81
**Average GMFEs (range)**						AUC		**2.00 (1.16 – 3.42)**	
						C_{max}		**1.96 (1.91 – 3.13)**	
**Average MRDs (range)**						AUC		**1.96 (1.01 – 1.93)**	
						C_{max}		**1.95 (1.20 – 1.76)**	
Hasselstrom et al., 1990 [19]	Cirrhosis	27-72	0.14	8 subjects x 50 trials	10 IR	AUC (nM•h)	517	381	0.74
						C_{max} (nM)	103	113	1.10
**GMFEs**						AUC		**2.30**	
						C_{max}		**1.60**	
**MRDs**						AUC		**1.35**	
						C_{max}		**1.1**	
Hoskin et al., 1989 [14]	Healthy	26-40	0.7	6 subjects x 67 trials	7.52 CR	AUC (ng•h/mL)	20.4	20.9	1.02
						C_{max} (ng/mL)	3.7	3.87	1.05
Preechagoon et al., 2010 [21]	Healthy	25-40	0	15 subjects x 27 trials	22.56 CR	AUC (ng•h/mL)	111	65.5	0.59
						C_{max} (ng/mL)	14.2	10.3	0.73
Kaiko et al., 1992 [22]	Healthy	25-40	0	14 subjects x 29 trials	22.56 CR	AUC (ng•h/mL)	61.3	65.5	1.07
						C_{max} (ng/mL)	9.7	10.3	1.06
Drake et al., 1996 [23]	Healthy	25-40	0	17 subjects x 24 trials	22.56 CR	AUC (ng•h/mL)	68.5	63.4	0.93
						C_{max} (ng/mL)	10.7	10.0	0.93
Kotb et al., 2005 [24]	Healthy	25-40	0.5	10 subjects x 40 trials	22.56 CR	AUC (ng•h/mL)	92.6	62.1	0.67
						C_{max} (ng/mL)	12.8	11.4	0.89
**Average GMFEs (range)**						AUC		**2.00 (1.23 – 3.01)**	

						C_{max}		**1.70 (1.40 – 2.34)**	
**Average MRDs (range)**						AUC		**1.95 (1.02 – 1.70)**	
						C_{max}		**1.42 (1.05 – 1.37)**	
Kotb et al., 2005 [24]	Cirrhosis	50-70	0.5	8 subjects x 50 trials	22.56 CR	AUC (ng•h/mL)	416	233	0.56
						C_{max} (ng/mL)	52.7	39.9	0.76
**GMFEs**						AUC		**3.16**	
						C_{max}		**2.21**	
**MRDs**						AUC		**1.76**	
						C_{max}		**1.32**	
Meineke et al., 2002 [25]	Cancer	19 - 65	0.56	9 subjects x 45 trials	0.5 mg/kg IV	CSF_{max} (ng/mL)	20.4	20.8	1.02
**GMFE**						CSF_{max}		**1.24**	
**MRD**						CSF_{max}		**1.02**	

Abbreviations: GMFE – Geometric mean fold error; MRD – Mean relative deviation; Obs – observed; Pred – Predicted; # females – proportion of females in the study; Pred/Obs – predicted/observed ratio; IV – intravenous; IR – immediate release; CR – controlled release; AUC – area under the plasma concentration – time curve; C_{max} – maximum plasma concentration.

[a] Actual free-based dose.

[b] AUC was not reported for this study, so clearance was used as a surrogate for model accuracy.

Supplemental Table 4. PBPK model-predicted and observed (mean and 90% CI) CBD exposure in healthy and cirrhotic adults after single intravenous or oral dose of CBD.

References	Population	Age (range)	# females	Population Size	Dose (mg) & Route	Parameters	Obs	Pred	Pred/Obs
Ohlsson et al., 1986 [26]	Healthy	19-33	0	5 subjects x 80 trials	20 IV	AUC (ng•h/mL)	311	375	1.20
**GMFE**						AUC		**1.91**	
**MRD**						AUC		**1.20**	
Taylor et al., 2018 [27]	Healthy	19-31	0.67	12 subjects x 34 trials	1,500 oral[a]	AUC (ng•h/mL)	1987	1416	0.71
						C_{max} (ng/mL)	335	239	0.71
Taylor et al., 2018 [27]	Healthy	23-29	0.83	6 subjects x 67 trials	1,500 oral[a]	AUC (ng•h/mL)	1517	1440	0.95
						C_{max} (ng/mL)	292	246	0.84
Taylor et al., 2018 [27]	Healthy	20-30	0.5	6 subjects x 67 trials	3,000 oral[a]	AUC (ng•h/mL)	2669	3154	1.18
						C_{max} (ng/mL)	533	524	0.98
Taylor et al., 2018 [27]	Healthy	18-34	1	6 subjects x 67 trials	4,500 oral[a]	AUC (ng•h/mL)	3215	3734	1.16
						C_{max} (ng/mL)	722	585	0.81
Taylor et al., 2018 [27]	Healthy	20-26	0.67	6 subjects x 67 trials	6,000 oral[a]	AUC (ng•h/mL)	3696	4655	1.26
						C_{max} (ng/mL)	782	785	1.00
**Average GMFEs (range)**						AUC		**1.87 (1.52 – 2.07)**	
						C_{max}		**1.59 (1.00 – 2.00)**	
**Average MRDs (range)**						AUC		**1.49 (1.08 – 1.26)**	
						C_{max}		**1.35 (1.00 – 1.23)**	
Taylor et al., 2019 [28]	Healthy	50-65	0.5	8 subjects x 50 trials	200 oral[b]	AUC (ng•h/mL)	449	442	0.98
						C_{max} (ng/mL)	148	129	0.87

Tayo et al., 2019 [28]	Healthy	48-65	0.625	8 subjects x 50 trials	200 oral[b]	AUC (ng•h/mL)	464	442	0.95
						C_{max} (ng/mL)	153	130	0.85
**Average GMFEs (range)**						AUC		**1.33 (1.24 – 1.41)**	
						C_{max}		**1.80 (1.76 – 1.84)**	
**Average MRDs (range)**						AUC		**1.06 (1.02 – 1.05)**	
						C_{max}		**1.24 (1.15 – 1.18)**	
Taylor et al., 2018 [27]	Healthy	20-38	0.44	9 subjects x 45 trials	1,500 B.I.D for 7 days	AUC Day 1 (ng•h/mL)	1444	1397	0.97
						C_{max} Day 1 (ng/mL)	362	321	0.89
						AUC Day 7 (ng•h/mL)	3236	3953	1.22
						C_{max} Day 7 (ng/mL)	541	394	0.73
**Average GMFEs (range)**						AUC		**1.67 (1.30 – 1.97)**	
						C_{max}		**2.00 (1.67 – 2.34)**	
**Average MRDs (range)**						AUC		**1.22 (1.03 – 1.22)**	
						C_{max}		**1.40 (1.12 – 1.37)**	
Taylor et al., 2019 [28]	Mild Cirrhosis	45-67	0.5	8 subjects x 50 trials	200 oral[b]	AUC (ng•h/mL)	648	750	1.16
						C_{max} (ng/mL)	233	180	0.77
**GMFEs**						AUC		**1.79**	
						C_{max}		**2.17**	
**MRDs**						AUC		**1.16**	
						C_{max}		**1.30**	
Taylor et al., 2019 [28]	Moderate Cirrhosis	46-60	0.375	8 subjects x 50 trials	200 oral[b]	AUC (ng•h/mL)	1054	1080	1.03
						C_{max} (ng/mL)	354	228	0.62
**GMFEs**						AUC		**1.30**	
						C_{max}		**2.86**	
**MRDs**						AUC		**1.03**	
						C_{max}		**1.61**	

Taylor et al., 2019 [28]	Severe Cirrhosis	45-65	0.5	6 subjects x 67 trials	200 oral[b]	AUC (ng•h/mL)	1855	1534	0.83
						C_{max} (ng/mL)	381	283	0.75
**GMFEs**						AUC		**1.93**	
						C_{max}		**2.26**	
**MRDs**						AUC		**1.20**	
						C_{max}		**1.33**	
**Average GMFEs (range)**						AUC		**1.70 (1.30 – 1.93)**	
						C_{max}		**2.43 (2.17 – 2.86)**	
**Average MRDs (range)**						AUC		**1.27 (1.03 – 1.20)**	
						C_{max}		**1.85 (1.30 – 1.61)**	

Abbreviations: B.I.D. – twice daily; GMFE – Geometric mean fold error; MRD – Mean relative deviation; Obs – observed; Pred – Predicted; # females – proportion of females in the study; Pred/Obs – predicted/observed ratio; AUC – area under the plasma concentration – time curve; C_{max} – maximum plasma concentration.

[a] Fasted

[b] Fed

Supplemental References

1. Zdrazil, B.; Felix, E.; Hunter, F.; Manners, E.J.; Blackshaw, J.; Corbett, S.; de Veij, M.; Ioannidis, H.; Lopez, D.M.; Mosquera, J.F.; et al. The ChEMBL Database in 2023: a drug discovery platform spanning multiple bioactivity data types and time periods. *Nucleic Acids Res* **2024**, *52*, D1180-D1192, doi:10.1093/nar/gkad1004.
2. Emoto, C.; Fukuda, T.; Johnson, T.; Neuhoff, S.; Sadhasivam, S.; Vinks, A. Characterization of Contributing Factors to Variability in Morphine Clearance Through PBPK Modeling Implemented With OCT1 Transporter. *CPT: Pharmacometrics & Systems Pharmacology* **2017**, *6*, 110-119, doi:10.1002/psp4.12144.
3. Rodgers, T.; Leahy, D.; Rowland, M. Physiologically based pharmacokinetic modeling 1: predicting the tissue distribution of moderate-to-strong bases. *J Pharm Sci* **2005**, *94*, 1259-1276, doi:10.1002/jps.20322.
4. Rodgers, T.; Rowland, M. Physiologically based pharmacokinetic modelling 2: predicting the tissue distribution of acids, very weak bases, neutrals and zwitterions. *J Pharm Sci* **2006**, *95*, 1238-1257, doi:10.1002/jps.20502.
5. Krekels, E.H.; Johnson, T.N.; den Hoedt, S.M.; Rostami-Hodjegan, A.; Danhof, M.; Tibboel, D.; Knibbe, C.A. From Pediatric Covariate Model to Semiphysiological Function for Maturation: Part II-Sensitivity to Physiological and Physicochemical Properties. *CPT Pharmacometrics Syst Pharmacol* **2012**, *1*, e10, doi:10.1038/psp.2012.12.
6. Osborne, R.; Joel, S.; Trew, D.; Slevin, M. Morphine and metabolite behavior after different routes of morphine administration: demonstration of the importance of the active metabolite morphine-6-glucuronide. *Clin Pharmacol Ther* **1990**, *47*, 12-19, doi:10.1038/clpt.1990.2.
7. Tzvetkov, M.V.; dos Santos Pereira, J.N.; Meineke, I.; Saadatmand, A.R.; Stingl, J.C.; Brockmöller, J. Morphine is a substrate of the organic cation transporter OCT1 and polymorphisms in OCT1 gene affect morphine pharmacokinetics after codeine administration. *Biochem Pharmacol* **2013**, *86*, 666-678, doi:10.1016/j.bcp.2013.06.019.
8. Verscheijden, L.F.M.; Litjens, C.H.C.; Koenderink, J.B.; Mathijssen, R.H.J.; Verbeek, M.M.; de Wildt, S.N.; Russel, F.G.M. Physiologically based pharmacokinetic/pharmacodynamic model for the prediction of morphine brain disposition and analgesia in adults and children. *PLoS Comput Biol* **2021**, *17*, e1008786, doi:10.1371/journal.pcbi.1008786.
9. Samara, E.; Bialer, M.; Mechoulam, R. Pharmacokinetics of cannabidiol in dogs. *Drug Metab Dispos* **1988**, *16*, 469-472.
10. Bansal, S.; Paine, M.F.; Unadkat, J.D. Comprehensive Predictions of Cytochrome P450 (P450)-Mediated In Vivo Cannabinoid-Drug Interactions Based on Reversible and Time-Dependent P450 Inhibition in Human Liver Microsomes. *Drug Metab Dispos* **2022**, *50*, 351-360, doi:10.1124/dmd.121.000734.
11. Bansal, S.; Ladumor, M.K.; Paine, M.F.; Unadkat, J.D. A Physiologically-Based Pharmacokinetic Model for Cannabidiol in Healthy Adults, Hepatically-Impaired Adults, and Children. *Drug Metabolism and Disposition* **2023**, *51*, 743-752, doi:10.1124/dmd.122.001128.
12. Tayo, B.; Taylor, L.; Sahebkar, F.; Morrison, G. A Phase I, Open-Label, Parallel-Group, Single-Dose Trial of the Pharmacokinetics, Safety, and Tolerability of Cannabidiol in Subjects with Mild to Severe Renal Impairment. *Clin Pharmacokinet* **2020**, *59*, 747-755, doi:10.1007/s40262-019-00841-6.

13. Bansal, S.; Maharao, N.; Paine, M.F.; Unadkat, J.D. Predicting the Potential for Cannabinoids to Precipitate Pharmacokinetic Drug Interactions via Reversible Inhibition or Inactivation of Major Cytochromes P450. *Drug Metab Dispos* **2020**, *48*, 1008-1017, doi:10.1124/dmd.120.000073.
14. Hoskin, P.J.; Hanks, G.W.; Aherne, G.W.; Chapman, D.; Littleton, P.; Filshie, J. The bioavailability and pharmacokinetics of morphine after intravenous, oral and buccal administration in healthy volunteers. *Br J Clin Pharmacol* **1989**, *27*, 499-505, doi:10.1111/j.1365-2125.1989.tb05399.x.
15. Hasselström, J.; Säwe, J. Morphine pharmacokinetics and metabolism in humans. Enterohepatic cycling and relative contribution of metabolites to active opioid concentrations. *Clin Pharmacokinet* **1993**, *24*, 344-354, doi:10.2165/00003088-199324040-00007.
16. Stuart-Harris, R.; Joel, S.P.; McDonald, P.; Currow, D.; Slevin, M.L. The pharmacokinetics of morphine and morphine glucuronide metabolites after subcutaneous bolus injection and subcutaneous infusion of morphine. *Br J Clin Pharmacol* **2000**, *49*, 207-214, doi:10.1046/j.1365-2125.2000.00141.x.
17. Lötsch, J.; Weiss, M.; Kobal, G.; Geisslinger, G. Pharmacokinetics of morphine-6-glucuronide and its formation from morphine after intravenous administration. *Clin Pharmacol Ther* **1998**, *63*, 629-639, doi:10.1016/S0009-9236(98)90086-8.
18. Lötsch, J.; Skarke, C.; Schmidt, H.; Liefhold, J.; Geisslinger, G. Pharmacokinetic modeling to predict morphine and morphine-6-glucuronide plasma concentrations in healthy young volunteers. *Clin Pharmacol Ther* **2002**, *72*, 151-162, doi:10.1067/mcp.2002.126172.
19. Hasselström, J.; Eriksson, S.; Persson, A.; Rane, A.; Svensson, J.O.; Säwe, J. The metabolism and bioavailability of morphine in patients with severe liver cirrhosis. *Br J Clin Pharmacol* **1990**, *29*, 289-297, doi:10.1111/j.1365-2125.1990.tb03638.x.
20. Masood, A.R.; Thomas, S.H. Systemic absorption of nebulized morphine compared with oral morphine in healthy subjects. *Br J Clin Pharmacol* **1996**, *41*, 250-252, doi:10.1111/j.1365-2125.1996.tb00192.x.
21. Preechagoon, D.; Sumyai, V.; Chulavatnatol, S.; Kulvanich, P.; Tessiri, T.; Tontisirin, K.; Pongjanyakul, T.; Uchaipichat, V.; Aumpon, S.; Wongvipaporn, C. Formulation development of morphine sulfate sustained-release tablets and its bioequivalence study in healthy Thai volunteers. *AAPS PharmSciTech* **2010**, *11*, 1449-1455, doi:10.1208/s12249-010-9518-5.
22. Kaiko, R.F.; Fitzmartin, R.D.; Thomas, G.B.; Goldenheim, P.D. The bioavailability of morphine in controlled-release 30-mg tablets per rectum compared with immediate-release 30-mg rectal suppositories and controlled-release 30-mg oral tablets. *Pharmacotherapy* **1992**, *12*, 107-113.
23. Drake, J.; Kirkpatrick, C.T.; Aliyar, C.A.; Crawford, F.E.; Gibson, P.; Horth, C.E. Effect of food on the comparative pharmacokinetics of modified-release morphine tablet formulations: Oramorph SR and MST Continus. *Br J Clin Pharmacol* **1996**, *41*, 417-420, doi:10.1046/j.1365-2125.1996.32810.x.
24. Kotb, H.I.; El-Kady, S.A.; Emara, S.E.; Fouad, E.A.; El-Kabsh, M.Y. Pharmacokinetics of controlled release morphine (MST) in patients with liver carcinoma. *Br J Anaesth* **2005**, *94*, 95-99, doi:10.1093/bja/aei007.
25. Meineke, I.; Freudenthaler, S.; Hofmann, U.; Schaeffeler, E.; Mikus, G.; Schwab, M.; Prange, H.W.; Gleiter, C.H.; Brockmöller, J. Pharmacokinetic modelling of morphine, morphine-3-glucuronide and morphine-6-glucuronide in plasma and cerebrospinal fluid of neurosurgical patients after short-term infusion of morphine. *Br J Clin Pharmacol* **2002**, *54*, 592-603, doi:10.1046/j.1365-2125.2002.t01-1-01689.x.

26. Ohlsson, A.; Lindgren, J.E.; Andersson, S.; Agurell, S.; Gillespie, H.; Hollister, L.E. Single-dose kinetics of deuterium-labelled cannabidiol in man after smoking and intravenous administration. *Biomed Environ Mass Spectrom* **1986**, *13*, 77-83, doi:10.1002/bms.1200130206.
27. Taylor, L.; Gidal, B.; Blakey, G.; Tayo, B.; Morrison, G. A Phase I, Randomized, Double-Blind, Placebo-Controlled, Single Ascending Dose, Multiple Dose, and Food Effect Trial of the Safety, Tolerability and Pharmacokinetics of Highly Purified Cannabidiol in Healthy Subjects. *CNS Drugs* **2018**, *32*, 1053-1067, doi:10.1007/s40263-018-0578-5.
28. Taylor, L.; Crockett, J.; Tayo, B.; Morrison, G. A Phase 1, Open-Label, Parallel-Group, Single-Dose Trial of the Pharmacokinetics and Safety of Cannabidiol (CBD) in Subjects With Mild to Severe Hepatic Impairment. *The Journal of Clinical Pharmacology* **2019**, *59*, 1110-1119, doi:10.1002/jcph.1412.
